# Controlling Nanoscale Heat With Optically Coupled Plasmonic Systems

**DOI:** 10.1002/nap2.70167

**Published:** 2026-06-13

**Authors:** José Luis Montaño‐Priede, Marek Grzelczak

**Affiliations:** ^1^ Centro de Física de Materiales (CFM‐MPC) CSIC‐UPV/EHU San Sebastián Spain; ^2^ Donostia International Physics Center (DIPC) San Sebastián Spain

**Keywords:** nanolenses, near‐field coupling, plasmon hybridization, reversible clustering, thermoplasmonics

## Abstract

The management of thermal effects in plasmonic nanostructures is frequently viewed as a detrimental byproduct rather than a useful controllable entity. Through numerical investigation of experimentally achievable systems, it is demonstrated how plasmon hybridization and near‐field coupling dictate the magnitude and spatial distribution of temperature, enabling precise spatiotemporal control over nanoscale heating. The results highlight the critical role of polarization and gap distance in tuning the thermal output of dimers, the ability of a trimer nanolens to focus heat into a sub‐diffraction volume reducing required laser intensities by an order of magnitude, and pronounced thermal differences in a switchable nanoparticle cluster. This work provides design principles for advanced thermoplasmonic systems where heat is not merely a detriment, but a dynamically controllable element for applications in catalysis, health, or active photonic devices.

## Graphical abstract

Optical coupling in plasmonic nanostructures is shown to enable precise spatiotemporal control of nanoscale heat. By engineering near‐field interactions in dimers, trimers, and switchable clusters, thermal energy is asymmetrically distributed, focused into sub‐diffraction volumes, or turned on/off via structural reconfiguration. These design principles transform heat from a detrimental byproduct into a controllable functional element.

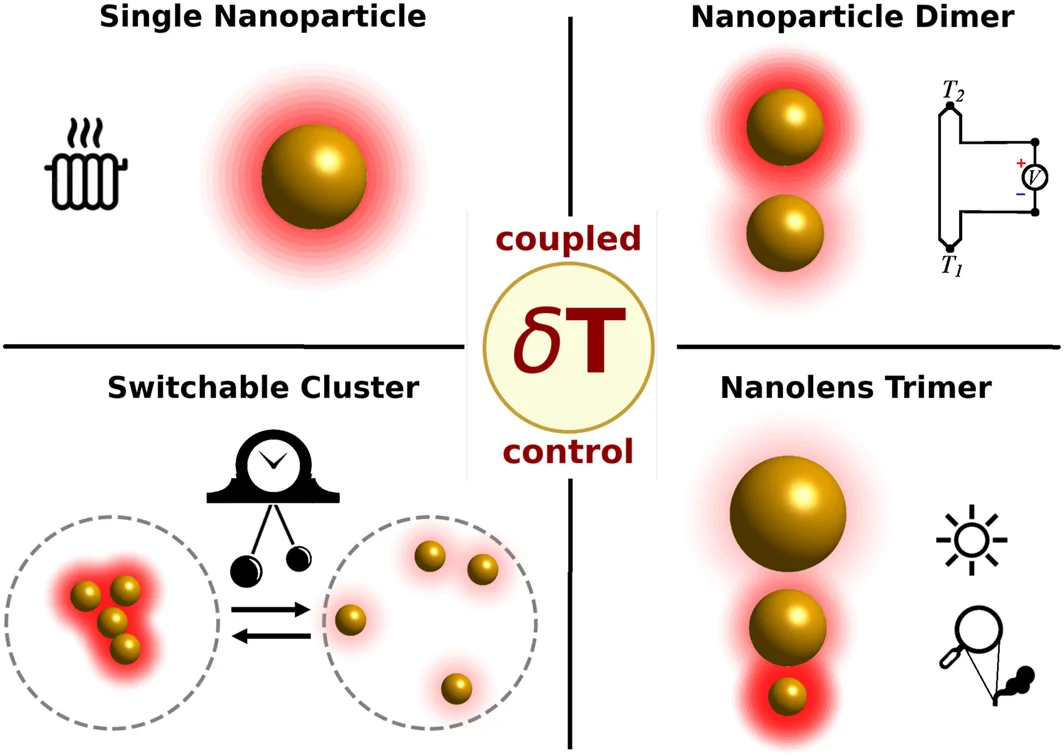

## 1 Introduction

Light–matter interaction in plasmonic nanomaterials [1, 2] gives rise to phenomena including surface‐enhanced Raman spectroscopy [3, 4], enhanced photoluminescence [5, 6], and colorimetric or refractive‐index sensing [4, 7]. These effects are significantly enhanced in the optically coupled regime, where small interparticle distances lead to plasmon hybridization and produce intensely localized electric fields not achievable with isolated particles [8, 9]. Over last decades, considerable effort has been dedicated to the precise geometrical engineering of such coupled nanostructures to tailor the optical response, controlling not only the magnitude of the field enhancement but also its spatial distribution and directionality [10, 11, 12].

Concurrently, the lossy nature of plasmonic nanoparticles results in inevitable heat dissipation via nonradiative pathways of plasmons [13], a phenomenon that is similarly amplified under optical coupling. This efficient light‐to‐heat conversion forms the foundation of thermoplasmonics and has been successfully harnessed in biomedical applications [14, 15] and catalysis [16, 17], where the design goal has traditionally been to maximize the total heat output [18, 19, 20, 21, 22, 23, 24, 25, 26]. However, a current shift in the field is moving beyond mere heat generation toward the precise spatiotemporal control of temperature at the nanoscale [15, 20]. For example, Chen et al. [27] engineered gold sphere‐rod nanodimers to spectrally match the solar irradiance spectrum for efficient solar thermal conversion, demonstrating control over the spectral absorption. Furthermore, Zundel et al. [28] showed that long‐range plasmonic coupling in bipartite nanoparticle array can be tailored to selectively heat one nanoparticle within the lattice unit cell, enabling controlled nanoscale thermal localization. Khosravi Khorashad et al. [29] further developed quantitative figures of merit for assessing thermal localization and heating efficiency in plasmonic systems under continuous‐wave (CW) excitation. These studies underscore that optical coupling between nanoparticles provides a powerful design principle for managing thermal gradients.

Inspired by existing experimentally realizable plasmonic constructs that have demonstrated significant optical functionality, we numerically explore their thermoplasmonic response to establish design principles for nanoscale heat control. We begin with the fundamental building blocks: single nanoparticles and dimers to establish baseline dependencies on size, gap, and polarization. We then investigate a nanolens trimer, a structure known for its ability to focus electromagnetic energy into deep subwavelength volumes [30, 31], and demonstrate that it similarly acts as a “nano‐heat‐lens”, concentrating thermal energy into a highly localized region. Finally, we extend our analysis to a reversibly clustering plasmonic system [32], evaluating the thermally distinct responses between aggregated and dispersed states. Our work provides a coherent framework for understanding and designing coupled plasmonic systems where heat generation is controllable, directional, and integral to the function of the system. This represents a conceptual shift from treating heat as a parasitic effect to be minimized toward leveraging it as a central design element.

## 2 Results

The optical and thermal properties of metallic nanoparticles are governed by the localized surface plasmon resonance (LSPR), a collective oscillation of the conduction electrons excited by an incident light at specific wavelength [33, 34]. Upon excitation, a portion of the absorbed energy is reradiated via scattering, whereas the remainder is dissipated nonradiatively, primarily as heat; this efficient light‐to‐heat conversion is the very basis of thermoplasmonics. Furthermore, since the LSPR is highly sensitive to the size, shape, material, and local environment of the nanoparticle, these parameters provide a direct means to control its thermal behavior.

The thermoplasmonic response of a single gold nanosphere (Figure 1) exhibits a strong size dependence. With increasing diameter, the LSPR redshifts (Figure 1a) and the absorption cross‐section intensifies (Supporting Information S1: Figure S1) as the dominant plasmonic mode transitions from dipole to higher‐order modes. Consequently, the resulting temperature rise is a function of both excitation wavelength and particle size. Figure 1b shows that the temperature increase reaches up to 60 K for diameters below 100 nm when excited at their respective absorption maxima $\left(\lambda_{\mathrm{abs}}\right)$ under an incident irradiance of $I_{0}=10^{9}$ ${\text{Wm}}^{-2}$. Contrary to the intuition that a larger metal volume invariably leads to more heating, the temperature increase does not scale monotonically. For diameters beyond approximately 80 nm, the temperature profile exhibits a nontrivial pattern of decreases and subsequent increases. This complex behavior is a direct consequence of the shifting dominance between different plasmonic modes.

**FIGURE 1:**
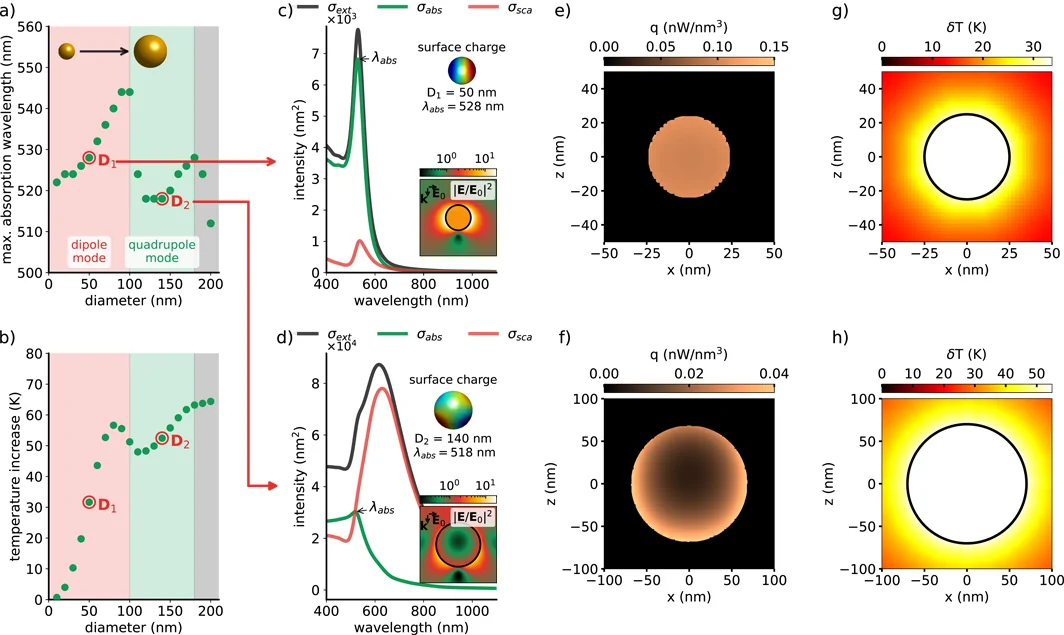
Thermoplasmonic response of an individual Au nanoparticle: Diameter dependence. (a) Maximum absorption wavelength $\left(\lambda_{\mathrm{abs}}\right)$ as a function of nanoparticle diameter. The shaded regions indicate the dominant plasmonic modes: dipole (red), quadrupole (green), and octupole (black). (b) Temperature increase (δ *T*) of the nanoparticle at $\lambda_{\mathrm{abs}}$ as a function of its diameter (excitation irradiance: $10^{9}$ ${\text{Wm}}^{-2}$). (c, d) Extinction, absorption, and scattering cross‐section spectra for nanoparticles with diameters of 50 nm (c) and 140 nm (d). Insets show the corresponding surface charge distribution and electric field maps in the plane of vibration (defined by the wave vector and E‐field polarization) at $\lambda_{\mathrm{abs}}$. (e, f) Heat power density (q) maps in the plane of vibration for the 50‐nm (e) and 140‐nm (f) nanoparticles. (g, h) Steady‐state temperature profiles for the 50‐nm (g) and 140‐nm (h) nanoparticles.

The nature of the dominant plasmonic mode also dictates the spatial distribution of the enhanced electric field in the near‐field of the nanoparticle (Supporting Information S1: Figure S2), a critical factor that influences the plasmonic coupling with neighboring nanostructures. Then, the temperature behavior is also affected by the distance, positions, and composition. Furthermore, the efficiency of heat generation is intrinsically linked to the balance between absorption and scattering. For a small nanoparticle (50 nm, Figure 1c) dominated by the dipole mode, absorption prevails over scattering at the LSPR, making it a highly efficient nano‐heater. Quantitatively, at its absorption maximum, the absorption efficiency is $\sigma_{\mathrm{abs}}/\left(\sigma_{\mathrm{abs}}+\sigma_{\mathrm{sca}}\right)=0.88$, meaning 88% of the extinguished light is converted to heat. In contrast, for a larger nanoparticle (140 nm, Figure 1d), scattering becomes the dominant decay pathway, with an absorption efficiency of only 0.50 at $\lambda_{\mathrm{abs}}$, significantly reducing its heating efficiency. From an application perspective, the size‐dependent absorption efficiency implies that smaller nanoparticles are preferred for photothermal therapy, where off‐target heating must be minimized and low irradiance is desirable to avoid photodamage. It is crucial to note that the wavelength of maximum extinction, commonly measured in UV‐Vis‐NIR spectroscopy, does not always coincide with the wavelength of maximum absorption, where the peak of temperature occurs. This underscores the necessity of numerical calculations or advanced experimental measurements to determine the optimal heating conditions in complex systems.

The heat power density profile within the nanoparticle reflects the underlying electric field distribution (Figure 1e,f; insets of Figure 1c,d). Although the 50‐nm sphere exhibits a relatively homogeneous heat source, the 140‐nm sphere shows a highly nonuniform profile concentrated at its surface. Nevertheless, under the uniform‐temperature approximation, the nanoparticle itself is considered isothermal with the resulting steady‐state temperature field decaying radially outward from its surface (Figure 1g,h). Although we focus on gold here, similar size‐dependent trends are observed for silver nanoparticles (Supporting Information S1: Figure S3), albeit with LSPRs shifted to lower wavelengths due to their different dielectric function.

Next, we investigate dimers comprising 50‐nm gold nanospheres. Such structures can be precisely assembled using bottom–up techniques, such as DNA origami scaffolds, which enable controlled nanoscale gaps (see Figure 2a, inset) [35, 36]. Although the high symmetry of a single sphere makes its response polarization‐independent, the dimer geometry breaks spherical symmetry, resulting in an axisymmetric structure ($D_{\infty h}$ point group). Consequently, its optical and thermal response strongly depends on the direction of the incident electric field relative to the dimer axis. We systematically examine how the thermoplasmonic response is modulated by the interparticle gap distance for the three key orthogonal polarization directions: transverse to the dimer axis, longitudinal along the dimer axis, and at $90^{0}$ orientation to the axis as illustrated in Figure 2.

**FIGURE 2:**
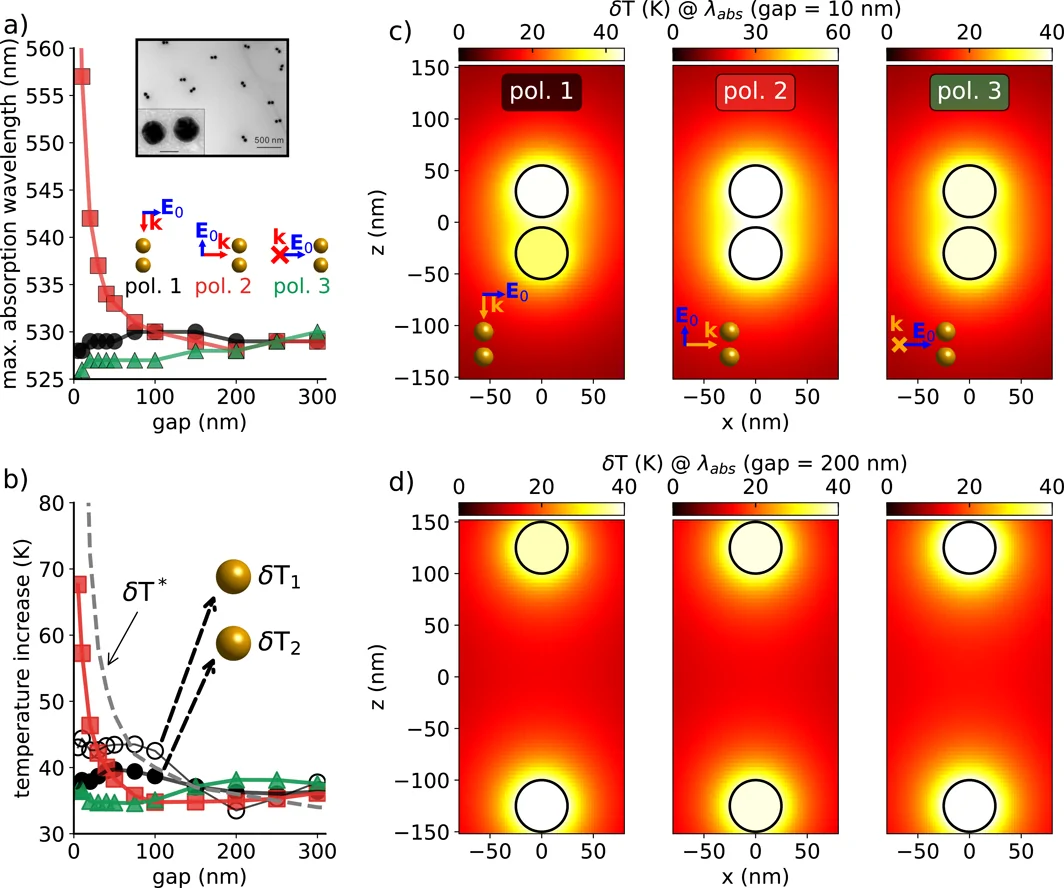
Thermoplasmonic response of 50‐nm Au nanoparticle dimers: Gap distance and polarization dependence. (a) Maximum absorption wavelength as a function of interparticle gap for three E‐field polarizations: transverse to the dimer axis (pol. 1), longitudinal along the dimer axis (pol. 2), and at $90^{0}$ orientation to the axis (pol. 3). The inset shows an illustrative TEM micrograph of dimers enabled by the DNA origami technique (reproduced from Ref. [35]; Copyright 2019 American Chemical Society). (b) Temperature increase of each nanoparticle ($\delta T_{1}$, solid marks, and $\delta T_{2}$, hollow marks) at $\lambda_{\mathrm{abs}}$ as a function of the gap for each polarization (as indicated in panel (a)). $\delta T^{*}=Q\left(1+a/d\right)/4\pi \kappa_{m}a$ (dashed line) is an approximation of collective temperature of each nanoparticle with radius a and gap d delivering equal heat power Q (calculated for a single nanoparticle, see Ref. [13]). Panels (a) and (b) show numerical simulation results for an excitation irradiance of $10^{9}$ ${\text{Wm}}^{-2}$. (c, d) Steady‐state temperature profiles for dimers with gaps of 10 nm (c) and 200 nm (d).

The optical coupling in a dimer system is modulated by its interparticle gap and the incident polarization, which together control the coupling strength and the hybridized plasmon mode. As illustrated in Figure 2a, the maximum absorption wavelength $\left(\lambda_{\mathrm{abs}}\right)$ for large gaps approaches the single nanoparticle resonance, almost polarization‐independent, confirming the absence of significant coupling. As the gap decreases, the resonance behavior diverges sharply based on polarization. For the transverse (Pol. 1) and $90^{0}$ (Pol. 3) polarizations, a slight blue‐shift of $\lambda_{\mathrm{abs}}$ is observed. In contrast, the longitudinal polarization (Pol. 2) induces a strong red‐shift, which becomes pronounced for gaps below 50 nm. This is a classic signature of plasmon hybridization, where the longitudinal excitation forms a bonding dipole plasmon mode [8]. The complete optical cross‐section spectra (Supporting Information S1: Figure S5) reveal that for Pol. 1 and Pol. 3, absorption remains the dominant process over scattering even at small gaps. However, for Pol. 2, the scattering cross‐section increases significantly at small gaps.

This polarization‐dependent coupling directly dictates the thermal response. Figure 2b plots the temperature increase of each nanoparticle ($\delta T_{1}$, $\delta T_{2}$) at their respective $\lambda_{\mathrm{abs}}$ as a function of the gap. For large gaps, the temperature for all polarizations converges to the single‐particle value of ∼33 K. As the gap decreases, the temperature generally rises due to the proximity of the two heat sources. However, for the longitudinally polarized case (Pol. 2) at sub‐50‐nm gaps, the temperature increases dramatically. This trend is compared to an approximated model, $\delta T^{*}$ (dashed line in Figure 2b) [19], which calculates the temperature by summing the contributions of individual noncoupled nanoparticles. The observed deviation, where $\delta T^{*}$ overestimates the actual temperature, arises because the model fails to account for the increased radiative scattering in the strongly coupled dimer.

A key finding is the emergence of asymmetric heating under transverse excitation (Pol. 1), whereas the other two polarizations do not produce significant temperature differences between nanoparticles. Under Pol. 1, the top nanoparticle ($\delta T_{1}$) consistently reaches a higher temperature than the bottom one ($\delta T_{2}$), with a difference of approximately ∼5 K at a 10‐nm gap (Figure 2c). This asymmetry persists, though inverted, at a 200‐nm gap (∼1.5 K, Figure 2d), indicating a complex interplay between near‐field shadowing and far‐field interference that is dominant under this transverse polarization. The experimental observability of this asymmetric heating relies on the precise control over size, gap distance, and polarization [19], all achievable with existing chemical synthesis, DNA origami scaffolds, and polarized illumination. Although dispersity in particle size or gap distance, as well as slight misalignments, can quantitatively modify the temperature ratio, the effect is still expected to be observable under realistic conditions as the temperature asymmetry has been shown to vary smoothly with these parameters [19]. Instead, these parameters can be tuned to make the asymmetric heating a controllable thermoplasmonic design element for precise devices. Ultimately, this establishes a localized thermal gradient that could, in principle, be harnessed in electrically‐connected systems to induce a Seebeck‐like thermoelectric response, pointing toward applications in plasmon‐enabled energy conversion.

The underlying mechanisms for these thermal profiles are revealed by the electric field and charge density distributions (Supporting Information S1: Figures S6 and S7 for a small gap and S8 and S9 for large gap). For a small gap under Pol. 1, the incident wave (k‐vector from top) induces a strong dipolar mode in the top nanoparticle, which effectively “shadows” the bottom particle. This leads to the formation of a higher‐order mode (quadrupole) in the bottom nanoparticle as confirmed by the charge density. The resultant higher absorption in the top nanoparticle explains the observed temperature asymmetry. For Pol. 2 at a small gap, the characteristic bonding dipole plasmon mode is established, concentrating immense electric field intensity in the gap and leading to strongly enhanced local heating. Regarding Pol. 3 at a small gap, the dipoles oscillate in‐phase in an unbounded configuration. The resulting charge screening between the nanoparticles suppresses near‐field coupling, leading to absorbed fields and temperature increases comparable to the large‐gap scenario. When the gap is large, the optical coupling vanishes, and both particles exhibit simple uncoupled dipolar excitations with temperatures similar to single nanoparticles.

In summary, the dimer system demonstrates that optical coupling introduces critical degrees of freedom, namely, polarization and gap distance. These allow for the tuning of the resonance wavelength and provide control over the magnitude and spatial distribution of heat generation.

The principles of asymmetric and enhanced heating established in the dimer structure can be strategically engineered in more complex architectures. We now explore a plasmonic nanolens, a structure recognized for its ability to focus electromagnetic energy into deep sub‐wavelength volumes, far beyond the diffraction limit. The concept, originally proposed as a self‐similar chain of metal nanospheres by Li et al. [30], relies on cascaded near‐field interactions to concentrate light. Such structures have been successfully realized experimentally, for example, through electrostatic self‐assembly methods [31] as shown in Figure 3a.

**FIGURE 3:**
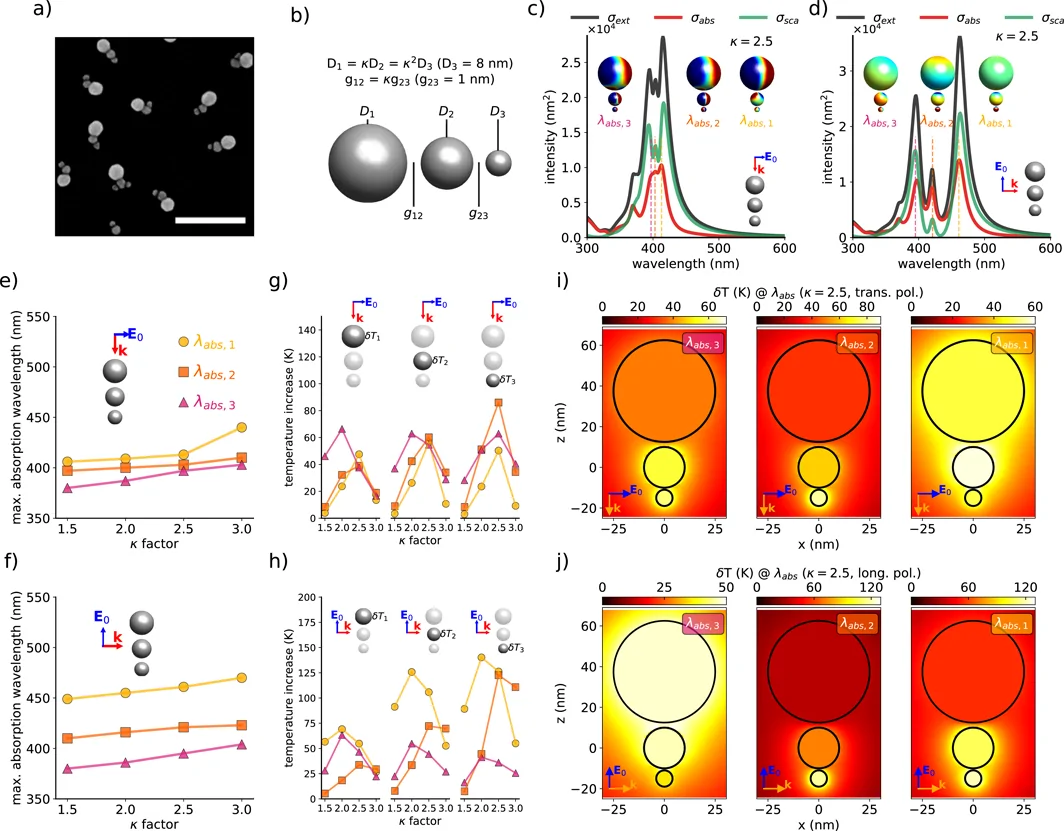
Thermoplasmonic response of Ag nanoparticle nanolens: Size ratio and polarization dependence. (a) Experimental TEM micrograph of trimer nanolenses (reproduced with permission from Ref. [31]; Copyright 2017 American Chemical Society). (b) Scheme of the self‐similar nanolens, where $D_{1}$, $D_{2}$ and $D_{3}$ are the nanoparticle diameters, $g_{12}$ and $g_{23}$ are the gap between $D_{1}$–$D_{2}$ and $D_{2}$–$D_{3}$, respectively, and κ is the self‐similar factor. All the results are for fixed $D_{3}=8$ nm and $g_{23}=1$ nm. (c, d) Extinction, absorption, and scattering cross‐section spectra of a nanolens with κ=2.5 under transverse (c) and longitudinal (d) excitations. Insets: Surface charge distributions at the three principal absorption resonances $\lambda_{\mathrm{abs,i}}$ (i=1,2,3 as indicated by the vertical dashed lines). (e, f) Evolution of the resonance absorption wavelengths with the self‐similar factor κ for transverse (e) and longitudinal (f) polarizations. (g, h) Temperature increase of individual nanoparticles ($\delta T_{1}$, $\delta T_{2}$, and $\delta T_{3}$) at $\lambda_{\mathrm{abs,i}}$ as a function of κ, for both transverse (g) and longitudinal (h) polarizations. Excitation irradiance: $10^{9}$ ${\text{Wm}}^{-2}$. (i, j) Steady‐state temperature profiles for nanolens with κ=2.5 at $\lambda_{\mathrm{abs,i}}$ under transverse (i) and longitudinal (j) polarizations.

This system allows us to investigate whether such field focusing translates into a “nano‐heat‐lens” effect, concentrating thermal energy into a deeply sub‐wavelength volume. To explore this, we investigate a silver nanolens trimer. Silver is chosen over gold for this study due to its lower ohmic losses, which results in narrower more distinguishable resonance peaks essential for resolving the complex coupling in this system. We model a linear trimer with geometrically scaled dimensions (see Figure 3b): the diameters follow $D_{1}=\kappa D_{2}=\kappa^{2}D_{3}$, and the gaps follow $g_{12}=\kappa g_{23}$, where $D_{3}=8$ nm and $g_{23}=1$ nm are fixed. The self‐similar factor κ is varied (1.5, 2, 2.5, 3) to systematically tune the optical response.

Given the axial symmetry of the linear trimer, we analyze the two principal incident polarizations: transverse and longitudinal to the chain axis. The extinction spectra for a nanolens with κ=2.5 (Figure 3c,d) reveal three distinct absorption peaks ($\lambda_{\mathrm{abs,1}}$, $\lambda_{\mathrm{abs,2}}$, $\lambda_{\mathrm{abs,3}}$) for each polarization, corresponding to hybridized plasmonic modes of the structure (see full cross‐section spectra in Supporting Information S1: Figure S10). As κ increases, the nanoparticles grow in size causing a systematic red‐shift of all resonance wavelengths (Figure 3e,f).

The thermoplasmonic response, characterized by the temperature increase of each nanoparticle at its respective $\lambda_{\mathrm{abs,i}}$, is complex and highly tunable (Figure 3g,h). A key trend is that temperatures generally increase with κ up to an optimum value of κ=2.5, beyond which they decrease due to a reduction in absorption efficiency mirroring the size‐effects observed in single nanoparticles. Although the absolute maximum temperature (∼140 K) occurs for κ=2 at $\lambda_{\mathrm{abs,1}}$ longitudinal polarization, we identify κ=2.5 as the optimal design parameter based on the most pronounced thermal gradient between nanoparticles, with temperatures of 122 K, 72 K, and 33 K for the small, medium, and large nanoparticles, respectively, at $\lambda_{\mathrm{abs,2}}$. For this optimal case, the smallest nanoparticle $\left(D_{3}\right)$ reaches a temperature approximately one order of magnitude higher (focusing factor ≈11.5) than a single isolated 8‐nm Ag sphere (see Equation 16 and Supporting Information S1: Table S1), quantitatively confirming the nano‐heat‐lens effect. Consequently, the required laser intensity to achieve the same heating effect could be reduced by a factor of ∼10, a crucial advantage for applications such as photothermal therapy or other thermally driven nanoscale processes.

The underlying coupling mechanisms are elucidated by the electric field and charge distributions for κ=2.5 (Supporting Information S1: Figures S11 and S12). Under transverse polarization, excitation at $\lambda_{\mathrm{abs,2}}$ induces a cascade of dipole‐mediated bonding modes that most effectively focus the electric field into the $g_{23}$ gap, yielding the highest temperature (∼86 K) in the smallest nanoparticle (Figure 3i). For longitudinal polarization, a similar but stronger effect is observed due to the direct field enhancement along the axis. Excitation at $\lambda_{\mathrm{abs,2}}$ establishes a fully coupled bonding dipole mode across all three nanoparticles, creating an intense hot spot and achieving a peak temperature of 122 K in $D_{3}$ (Figure 3j). It is worth noting that at this resonance, the absorption cross‐section dominates over scattering, resulting in a heating efficiency that is significantly higher than in the other modes, where scattering plays a more dominant role (Figure 3d). At other resonances, the mode hybridization is less effective at focusing energy exclusively into the smallest particle (as in the case of Figure 3d for $\lambda_{\mathrm{abs,1}}$ or $\lambda_{\mathrm{abs,3}}$), leading to reduced nanolens efficiency or a complete suppression of the effect. Preserving such steep thermal gradients under illumination requires pulsed excitation to avoid heat diffusion and gradient degradation (see discussion below).

In conclusion, the silver nanolens demonstrates that cascaded near‐field coupling can be engineered to concentrate not only electric fields but also thermal energy into a nanoscale volume creating an ultra‐localized heat source. The performance is highly sensitive to the geometric factor κ and the excitation wavelength, with an optimal configuration identified here. Navigating this multiparameter design space is challenging, but emerging algorithm‐driven optimization tools promise to accelerate the finding of optimal geometries for specific thermoplasmonic applications [37].

We now extend our study to a dynamic cluster of gold nanoparticles confined within a capsule, where the optical and thermal properties can be actively switched by external stimulus. The concept, illustrated in Figure 4, a switchable cluster under confinement.

**FIGURE 4:**
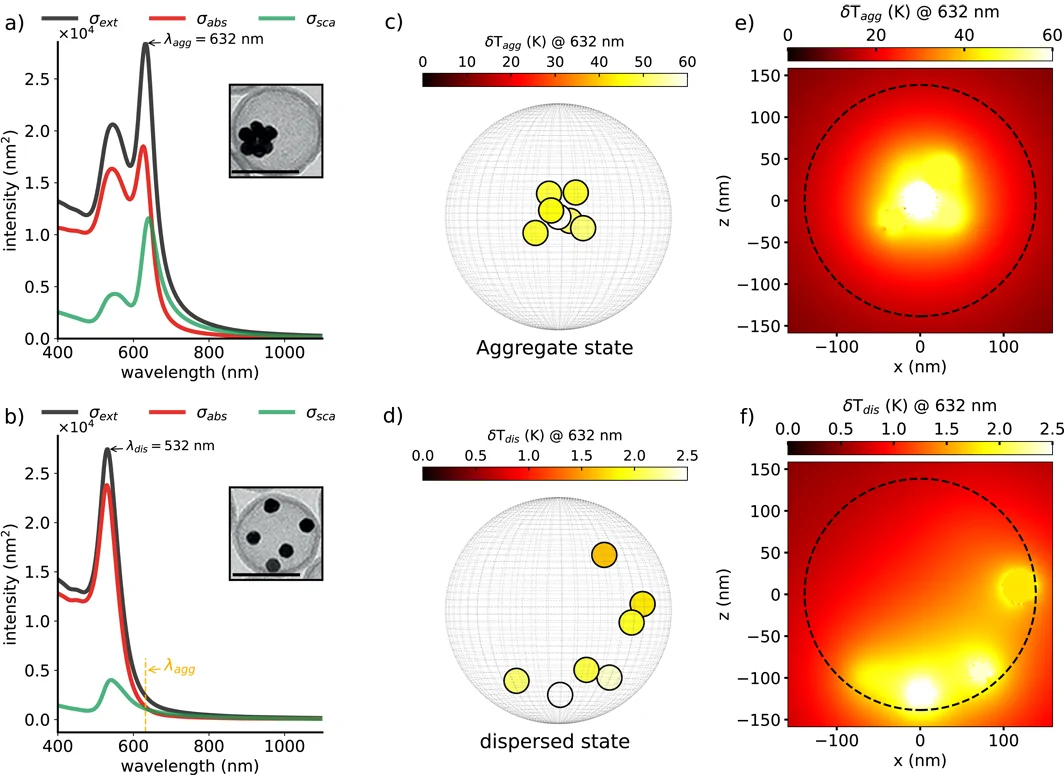
Thermoplasmonic response of gold nanoparticle clusters confined within a silica shell: Aggregated and dispersed states. (a, b) Extinction, absorption, and scattering cross‐section spectra for aggregated (a) and dispersed (b) nanoparticle arrangements. Each cluster consists of seven Au nanoparticles on average within a silica shell. The spectra result from averaging over 15 distinct random nanoparticle positions for each state. Insets show experimental TEM micrographs of Au nanoparticle clusters in the two states (adapted from Ref. [32]; reproduced with permission). (c, d) Increase of temperature of individual nanoparticles for representative aggregated (c) and dispersed (d) configurations, excited at $\lambda_{\mathrm{agg}}=632$ nm. Excitation irradiance: $10^{9}$ ${\text{Wm}}^{-2}$. (e, f) Steady‐state temperature profiles (*xz*‐plane at *y* = 0 nm) for the aggregated (e) and dispersed (f) states at $\lambda_{\mathrm{agg}}=632$ nm. Dashed black line indicates the shell inner surface.

The model is based on our past experimental systems of polystyrene‐functionalized gold nanoparticles encapsulated in a mesoporous silica shell permeable to solvent molecules [32]. The reversible clustering is driven by solvent composition, where low‐polarity solvent maintains nanoparticles dispersed whereas high‐polarity solvent leads to aggregation due to hydrophobic forces. Each cluster consists of seven Au nanoparticles (40 nm) within a silica shell of 20‐nm thick and inner diameter of 277 nm. Experimental TEM images of the aggregated and dispersed states are shown in the insets of Figure 4a,b, respectively. In our simulations, the aggregated state features a minimum interparticle gap of 2 nm. The optical cross‐section spectra (Figure 4a,b) reveal a broad red‐shifted extinction band at $\lambda_{\mathrm{agg}}=632$ nm for the aggregate state due to strong near‐field coupling, whereas the dispersed state shows a blue‐shifted band at $\lambda_{\mathrm{dis}}=532$ nm characteristic of uncoupled dipoles.

We calculated the thermal response under fixed excitation at $\lambda_{\mathrm{agg}}=632$ nm. Figure 4c shows the temperature distribution for a representative aggregated configuration, where the strong optical coupling leads to a significant temperature increase of approximately 60 K. In contrast, the same excitation of the dispersed state (Figure 4d) yields a temperature rise of ∼2.5 K per nanoparticle as the excitation of the system is far from resonance. This remarkable temperature difference between the two states corresponds to an enhancement factor of approximately 24 $\left(\delta T_{\mathrm{agg}}/\delta T_{\mathrm{dis}}\right)$, revealing the potential for a self‐regulating system where the optical and thermal states are intrinsically coupled. This factor is independent of the incident irradiance and reflects the purely structural and coupling‐dependent heating efficiency. The steady‐state temperature profiles (Figure 4e,f) further highlight this contrast, showing intense, localized heating in the aggregated core and a mild distributed temperature field in the dispersed case.

Although the localized thermal profiles presented here are calculated for an idealized steady‐state scenario (nanoparticles in an infinite homogeneous water medium), prolonged continuous illumination in a real finite system would lead to cumulative heat diffusion, eventually homogenizing the temperature field in the microenvironment, yielding to the degradation of thermal gradients. To preserve these focused thermal profiles (such as the intense heat in the smallest nanoparticle of the nanolens), the excitation must be applied in pulses shorter than the characteristic heat diffusion time. This timescale is inherently transient; for a nanoscale heat source in water, the thermal evolution occurs on the order of nanoseconds. Indeed, following the framework of Baffou [13], a plasmonic system can reach ∼84% of its final temperature increase in times as short as 10 ns, with a comparably rapid decay (e.g., 20–50 ns) once illumination ceases. As concluded by Khosravi Khorashad et al. [29], pulsed excitation provides stronger thermal localization than CW illumination, which suffers from heat spreading over long times. Therefore, our findings indicate that the experimental observations of the proposed here nano‐heat spots requires a pulsed excitation to balance incident power and pulse width. A higher incident power enables the target temperature to be reached faster, thereby permitting the use of shorter pulses to effectively confine the heat. Optimizing this pulse‐power interplay is essential to achieve intense localized heating while mitigating global heat accumulation in the microenvironment, constituting a key design principle for thermoplasmonics under optically coupled regime. A summary of the key design parameters and quantitative thermal metrics for each architecture is provided in Table 1.

**TABLE 1:** Design principles and quantitative thermal metrics for optically coupled plasmonic systems.

Architecture	Key design parameters	Thermal functionality	Quantitative metric
Single NP	Size, material, and excitation wavelength $\left(\lambda_{\mathrm{exc}}\right)$	Size‐tunable heating efficiency	Abs. efficiency = 0.88 (D=50 nm) to 0.50 (D=140 nm)
Dimer	Size, material, gap, polarization, and $\lambda_{\mathrm{exc}}$	Asymmetric heating (transverse pol.);	$\Delta T_{1}-\Delta T_{2}\;\approx \;5$ K at g=10 nm;
		Enhanced longitudinal heating (longitudinal pol.)	$\Delta T_{\text{max}}\;\approx \;60$ K at g=10 nm
Nanolens trimer	Size, material, gap, self‐similar factor, polarization, and $\lambda_{\mathrm{exc}}$	Heat focusing into sub‐diffraction volume (nano‐heat‐lens)	Photothermal focusing factor (PFF) ≈11.5 for κ=2.5, long. pol., $\lambda_{\text{abs},2}$
Switchable cluster	NP size, cluster size (NP amount), gap, and confinement (silica shell)	Binary on/off heating via structural reconfiguration	Enhancement factor $\delta T_{\text{agg}}/\delta T_{\text{dis}}\;\approx \;24$ at $\lambda_{\mathrm{exc}}=632$ nm

*Note:* All values calculated for $I_{0}=10^{9}$ W/$m^{2}$ in water.

## 3 Conclusion

In summary, inspired by experimentally achievable plasmonic nanostructures, we provided a numerical design principle for achieving precise spatiotemporal control over nanoscale heat generation under optical coupling. We have demonstrated that by engineering near‐field interactions (through parameters such as gap distance, polarization, and hierarchical geometry) it is possible to dictate not only the magnitude but also the localization and symmetry of photothermal heating. This control ranges from creating asymmetric thermal profiles in dimers, to quantifying a nano‐heat‐lens effect in trimers (where the smallest nanoparticle heats 1 order of magnitude more than an isolated sphere reducing required laser intensity), to demonstrating a large temperature difference (∼24 fold) under reversible states of nanoparticle clusters as a promising direction for future self‐regulating systems.

The implications of this work are significant for the future of nanoscale thermal management and actuation. The principles of controlled heating elucidated here, through symmetry breaking, mode hybridization, and structural reconfiguration, can be directly applied to challenges in photothermal catalysis, where precise temperature control at reaction sites could enhance selectivity and yield; in nanofabrication and nano‐soldering, where directed heat is required; and in the development of smart adaptive plasmonic devices. By providing a coherent stepwise rationale from fundamental single‐particle effects to complex active systems, this study lays the groundwork for designing next‐generation thermoplasmonic platforms where heat is not merely a waste, but a finely controlled design element, especially under an optically coupled regime.

## 4 Experimental Section

The thermoplasmonic response of the studied nanoparticle systems was modeled using a Green's function approach based on the Laplace Matrix Inversion (LMI) method developed by Baffou et al. [38] In this work, we systematize and generalize the LMI approach into a clear sequential computational procedure, making it more accessible for implementation and extension to multiparticle systems. This structured framework enables the efficient computation of steady‐state temperature distributions in complex plasmonic nanostructures under optical illumination from the electromagnetic response to the final thermal map.

### 4.1 Electromagnetic Response Calculation

The optical response of metallic nanoparticles under continuous plane‐wave illumination was computed using the boundary element method as implemented in the MNPBEM toolbox [39]. This approach solves Maxwell's equations for metallic nanoparticles embedded in dielectric environments by discretizing particle surfaces into boundary elements and computing surface charge and current distributions. The dielectric function of gold and silver was modeled using experimental data from Johnson and Christy [40]. The surrounding medium was water with dielectric constant of $\epsilon_{m}=1.777$. Since this work assumes local bulk permittivities, our simulations employ the classical local‐response approximation (LRA) [41]. For gaps as small as 1 nm, quantum effects, such as charge‐transfer processes due to electron tunneling, nonlocal screening, surface‐enabled Landau damping, can become relevant [41, 42, 43]. However, for noble metals (Au, Ag) and gap distances ≥ 1 nm, the classical LRA remains the standard framework for modeling plasmonic coupling and provides a reasonable semiquantitative description. Therefore, we expect the trends and relative enhancement factors to remain valid, although absolute temperatures at the smallest gap may be moderately overestimated.

The simulations were conducted in two stages. First, the extinction, scattering, and absorption cross‐section spectra were computed in the wavelength range of 500–1100 nm for gold nanoparticles and from 300 to 600 nm for silver nanoparticles.

Then, for each nanoparticle system, the electric field enhancement distribution $|E\left(r\right)/E_{0}{|}^{2}$ was calculated at the absorption maximum wavelength, $\lambda_{\text{abs}}$, where $|E_{0}{|}^{2}=2I_{0}/\epsilon_{0}n_{m}c$ is the incident field amplitude, $I_{0}=10^{9}$ ${\text{Wm}}^{-2}$ is the incident irradiance, $\epsilon_{0}$ is the vacuum permittivity, and c is the speed of light in vacuum. For this, the simulation domain was discretized using a total of N cubic cells of volume ΔV=ΔxΔyΔz each.

### 4.2 Heat Generation Calculation

A subset of M computational cells (M⊂N), distributed inside the P nanoparticles forming the systems, was selected. For each cell i∈M located at position $r_{i}$, the heat power density is calculated as follows [20]:

(1) $$q\left(r_{i}\right)=\frac{\omega I_{0}}{n_{m}c}\text{Im}\left[\epsilon \left(r_{i}\right)\right]{\left|\frac{E\left(r_{i}\right)}{E_{0}}\right|}^{2},$$

where $\omega =2\pi c/\lambda_{\text{abs}}$ is the angular frequency, $n_{m}=\sqrt{\epsilon_{m}}$ is the refractive index of the surrounding medium, and $\text{Im}\left[\epsilon \left(r_{i}\right)\right]$ is the imaginary part of the dielectric function at cell i.

The total heat power generated by the nanoparticle k (k∈P) is as follows:

(2) $$Q_{k}=\sum_{i\in M_{k}}q\left(r_{i}\right)\Delta V=\frac{\omega I_{0}\Delta V}{n_{m}c}\text{Im}\left(\epsilon_{k}\right)c_{k},$$

where $M_{k}$ denotes the set of cells belonging to the nanoparticle k and $c_{k}$ is the integrated field coefficient defined as:

(3) $$c_{k}=\sum_{i\in M_{k}}{\left|\frac{E\left(r_{i}\right)}{E_{0}}\right|}^{2}.$$

### 4.3 Temperature Calculation via LMI

The steady‐state temperature distribution was computed by solving the heat diffusion equation:

(4) $$\kappa_{m}\nabla^{2}T\left(r\right)=-q\left(r\right),$$

where $\kappa_{m}$ is the thermal conductivity of the medium ($\kappa_{H_{2}O}=0.598$ ${\text{Wm}}^{-1}K^{-1}$). For metallic nanoparticles in aqueous environments ($\kappa_{\text{Au}},\kappa_{\text{Ag}}\gg \kappa_{H_{2}O}$, with $\kappa_{\text{Au}}=318$ ${\text{Wm}}^{-1}K^{-1}$ and $\kappa_{\text{Ag}}=429$ ${\text{Wm}}^{-1}K^{-1}$), the temperature within each nanoparticle is approximately uniform, following the uniform temperature approximation (UTA) [19]. As shown by Baffou et al. [19], the UTA remains valid even under highly localized heating for nanoparticles with aspect ratios up to ∼10, a condition satisfied by all systems studied here. Importantly, the validity of the UTA depends only on the thermal conductivity contrast between the metal and the surrounding medium (here $\kappa_{\mathrm{metal}}/\kappa_{\mathrm{water}}>$ 500) not on the spatial distribution of the heat source; thus, strong optical coupling does not compromise this approximation.

To determine the temperature of each nanoparticles in a system, we employed the LMI formalism. Although the original work derived the formalism for a single structure and provided the specific coupled equations for a dimer (two disjointed structures), we generalize it here for an arbitrary number P of nanoparticles.

The uniform temperatures of the nanoparticles, represented by the vector T = [$T_{1},T_{2}$, $\text{…},T_{P}$], are obtained by solving the following coupled linear system derived from energy conservation and the LMI formalism:

(5) $$\left(\begin{array}{c} c_{1}\\ c_{2}\\ \text{⋮}\\ c_{k}\\ \text{⋮}\\ c_{P} \end{array}\right)=\frac{4\pi \kappa_{m}n_{m}c}{\omega I_{0}\text{Im}\left(\epsilon \right)\Delta V}\left(\begin{array}{cccccc} r_{L1} & r_{12} & \text{⋯} & r_{1l} & \text{⋯} & r_{1P}\\ r_{21} & r_{L2} & \text{⋯} & r_{2l} & \text{⋯} & r_{2P}\\ \text{⋮} & \text{⋮} & \text{⋱} & \text{⋮} & \text{⋮} & \text{⋮}\\ r_{k1} & r_{k2} & \text{⋯} & r_{Lk} & \text{⋯} & r_{kP}\\ \text{⋮} & \text{⋮} & \text{⋮} & \text{⋮} & \text{⋱} & \text{⋮}\\ r_{P1} & r_{P2} & \text{⋯} & r_{Pl} & \text{⋯} & r_{LP} \end{array}\right)\left(\begin{array}{c} T_{1}\\ T_{2}\\ \text{⋮}\\ T_{k}\\ \text{⋮}\\ T_{P} \end{array}\right),$$

where $r_{Lk}$ is the Laplace radius of the nanoparticle k and $r_{kl}$ is the coupled radius term between nanoparticles k and l (k≠l∈P) both radius defined as follows:

(6) $$r_{Lk}=\sum_{i,j\in M_{k}}{\left(A^{-1}\right)}_{ij},$$

(7) $$r_{kl}=\sum_{i\in M_{k}}\sum_{j\in M_{l}}{\left(A^{-1}\right)}_{ij}.$$

The Laplace matrix A is constructed with elements:

(8) $$A_{ij}=\left\{\begin{array}{cc} \frac{1}{R_{\text{eff}}}, & i=j\\ \frac{1}{\left|r_{i}-r_{j}\right|}, & i\neq j \end{array}\right.$$

where $R_{\text{eff}}={\left(3\Delta V/4\pi \right)}^{1/3}$ is the effective radius of a cubic cell. The matrix of radii in Equation (5) is symmetric with $r_{kl}=r_{lk}$ since A is symmetric (see Equation 8). For the case of P=2, the system Equation (5) reduces to the pair of equations (38) and (39) presented in Ref. [38]. Our formulation thus provides a direct and general framework to compute the temperature distribution in clusters composed of any number of optically and thermally interacting plasmonic nanoparticles.

### 4.4 Fictive Source Distribution and Temperature Reconstruction

To enforce uniform temperature within each nanoparticle, following the UTA, a fictive electric field enhancement distribution $e_{i}^{2}$ was computed that satisfies:

(9) $$e_{i}^{2}=\frac{4\pi \kappa_{m}n_{m}c}{\omega I_{0}\text{Im}\left(\epsilon \right)\Delta V}\sum_{j=1}^{M}{\left(A^{-1}\right)}_{ij}T_{k},$$

where $T_{k}$ is the uniform temperature of the nanoparticle that cell j belongs to as determined from the solution of the coupled system in Equation (5).

The complete temperature distribution over the i∈N cells was then reconstructed throughout the computational domain using the superposition principle:

(10) $$T\left(r_{i}\right)=\sum_{j=1}^{M}G\left(r_{i},r_{j}\right)\;q_{\text{fictive}}\left(r_{j}\right)\;\Delta V,$$

where the thermal Green's function is defined as follows:

(11) $$G\left(r_{i},r_{j}\right)=\left\{\begin{array}{cc} \frac{1}{4\pi \kappa_{m}r_{Lj}}, & r_{i}=r_{j}\\ \frac{1}{4\pi \kappa_{m}|r_{i}-r_{j}|}, & r_{i}\neq r_{j} \end{array}\right.$$

with $r_{Lj}$ being the Laplace radius that accounts for the self‐interaction term, and the fictive heat source density is as follows:

(12) $$q_{\text{fictive}}\left(r_{j}\right)=\frac{\omega I_{0}}{n_{m}c}\text{Im}\left(\epsilon \right)\;e_{j}^{2}.$$

The validity of this methodology was confirmed by reproducing key benchmark cases from the literature as shown in Supporting Information S1: Figure S13, along with key theoretical results such as the characteristic $r^{-1}$ temperature decay outside nanoparticles [19]. Although more fundamental approaches exist that model electron–phonon interactions and interface effects, including those characterized by the Kapitza length [44, 45], our approach provides reliable thermal predictions for complex plasmonic nanostructures.

### 4.5 Treatment of Silica‐Encapsulated Nanoparticle Clusters in Water

The cluster system consisted of seven gold nanoparticles encapsulated within a porous silica shell, with the entire system immersed in water, system experimentally achieved in Ref. [32].

Considering that water is the dominant continuous phase both inside and outside the silica shell and permeates through the porous silica, the effective optical and thermal properties were calculated with water as the matrix phase.

The effective dielectric constant for the silica‐water composite was calculated using Bruggeman's effective medium approximation [46], which provides a symmetric treatment of both components:

(13) $$\left(1-f\right)\frac{\epsilon_{H_{2}O}-\epsilon_{\text{eff}}}{\epsilon_{H_{2}O}+2\epsilon_{\text{eff}}}+f\frac{\epsilon_{{\text{SiO}}_{2}}-\epsilon_{\text{eff}}}{\epsilon_{{\text{SiO}}_{2}}+2\epsilon_{\text{eff}}}=0,$$

where $\epsilon_{H2O}=1.777$, $\epsilon_{{\text{SiO}}_{2}}=2.103$ (the value used in Ref. [32] corresponding to fused silica), and f is the volume fraction of silica in the cluster. For a shell with outer radius $R_{\text{outer}}=158.5$ nm and thickness t=20 nm, the silica volume fraction is as follows:

(14) $$f=\frac{R_{\text{outer}}^{3}-{\left(R_{\text{outer}}-t\right)}^{3}}{R_{\text{outer}}^{3}}\approx 0.33.$$

Solving Equation (13) numerically yields $\epsilon_{\text{eff}}\;\approx \;1.881$ for the effective medium surrounding the gold nanoparticles.

The effective thermal conductivity was calculated using Bruggeman's approximation to maintain consistency with the optical modeling:

(15) $$\left(1-f\right)\frac{\kappa_{H_{2}O}-\kappa_{\text{eff}}}{\kappa_{H_{2}O}+2\kappa_{\text{eff}}}+f\frac{\kappa_{{\text{SiO}}_{2}}-\kappa_{\text{eff}}}{\kappa_{{\text{SiO}}_{2}}+2\kappa_{\text{eff}}}=0,$$

where $\kappa_{{\text{SiO}}_{2}}=1.4$ ${\text{Wm}}^{-1}K^{-1}$ [47]. Solving Equation (15) numerically yields $\kappa_{\text{eff}}\approx 0.81$ ${\text{Wm}}^{-1}K^{-1}$, which is still much smaller than the thermal conductivities of the metallic nannoparticles, then the UTA is still valid.

### 4.6 Photothermal Focusing Factor

To quantify the heat concentration capability of the nanolens trimer, we define the *photothermal focusing factor* (PFF) as the ratio of the temperature increase of the smallest nanoparticle within the trimer structure to the temperature increase of an isolated single nanoparticle of identical size and material under identical illumination conditions. Mathematically, the PFF is expressed as follows:

(16) $$\text{PFF}=\frac{\delta T_{D3}^{\left(\text{trimer}\right)}}{\delta T_{\text{single}}}$$

where $\delta T_{D3}^{\left(\text{trimer}\right)}$ is the steady‐state temperature increase of the smallest nanoparticle (diameter $D_{3}=8$ nm) in the silver nanolens trimer and $\delta T_{\text{single}}$ is the temperature increase of an isolated (8 nm) silver nanosphere under the same incident irradiance ($I_{0}=10^{9}$ W/$m^{2}$) at its own absorption maximum ($\lambda_{\text{abs}}=383$ nm, δT=10.7 K).

This dimensionless metric provides a structure‐intrinsic measure of the thermal nanofocusing efficiency. A PFF greater than unity indicates that the near‐field coupling with the larger neighboring nanoparticles enhances the photothermal response of the smallest nanoparticle beyond its isolated‐particle value. Conversely, a PFF value below unity would indicate suppression of heating due to shielding (i.e., optical shadowing) or radiative losses.

## Author Contributions

**José Luis Montaño‐Priede:** conceptualization, methodology, software, data curation, investigation, validation, formal analysis, visualization, writing – original draft, writing – review and editing. **Marek Grzelczak:** conceptualization, methodology, data curation, investigation, validation, formal analysis, supervision, funding acquisition, project administration, resources, writing – original draft, writing – review and editing.

## Supporting information


Supporting Information S1

## Data Availability

The data that support the findings of this study are openly available in Zenodo at https://doi.org/10.5281/zenodo.18484667.

## References

[1] H. Yu , Y. Peng , Y. Yang , and Z.‐Y. Li , “Plasmon‐Enhanced Light–Matter Interactions and Applications,” npj Computational Materials 5, no. 1 (2019): 45, 10.1038/s41524-019-0184-1.

[2] L. Montaño‐Priede , O. Peña‐Rodríguez , A. Rivera , A. Guerrero‐Martínez , and U. Pal , “Optimizing the Electric Field Around Solid and Core–Shell Alloy Nanostructures for Near‐Field Applications,” Nanoscale 8, no. 31 (2016): 14836–14845, 10.1039/c6nr03801h. 27451969

[3] J. Langer , D. Jimenez de Aberasturi , J. Aizpurua , et al., “Present and Future of Surface‐Enhanced Raman Scattering,” ACS Nano 14, no. 1 (2020): 28–117, 10.1021/acsnano.9b04224. PMC6990571 31478375

[4] K. Kant , R. Beeram , Y. Cao , et al., “Plasmonic Nanoparticle Sensors: Current Progress, Challenges, and Future Prospects,” Nanoscale Horizons 9, no. 12 (2024): 2085–2166, 10.1039/d4nh00226a. PMC11378978 39240539

[5] D. Dahal , P. Ray , and D. Pan , “Unlocking the Power of Optical Imaging in the Second Biological Window: Structuring Near‐Infrared II Materials From Organic Molecules to Nanoparticles,” WIREs Nanomedicine and Nanobiotechnology 13, no. 6 (2021): e1734, 10.1002/wnan.1734. 34159753

[6] D. C. Mor , G. Aktug , K. Schmidt , et al., “Plasmon‐Enhanced Fluorescence (Bio)Sensors and Other Bioanalytical Technologies,” Trends in Analytical Chemistry 183 (2025): 118060, 10.1016/j.trac.2024.118060.

[7] M. Li , S. K. Cushing , and N. Wu , “Plasmon‐Enhanced Optical Sensors: A Review,” Analyst 140, no. 2 (2015): 386–406, 10.1039/c4an01079e. PMC4274271 25365823

[8] P. Nordlander , C. Oubre , E. Prodan , K. Li , and M. I. Stockman , “Plasmon Hybridization in Nanoparticle Dimers,” Nano Letters 4, no. 5 (2004): 899–903, 10.1021/nl049681c.

[9] N. J. Halas , S. Lal , W.‐S. Chang , S. Link , and P. Nordlander , “Plasmons in Strongly Coupled Metallic Nanostructures,” Chemical Reviews 111, no. 6 (2011): 3913–3961, 10.1021/cr200061k. 21542636

[10] P. Anger , P. Bharadwaj , and L. Novotny , “Enhancement and Quenching of Single‐Molecule Fluorescence,” Physical Review Letters 96, no. 11 (2006): 113002, 10.1103/physrevlett.96.113002. 16605818

[11] J. T. Hugall , A. Singh , and N. F. van Hulst , “Plasmonic Cavity Coupling,” ACS Photonics 5, no. 1 (2018): 43–53, 10.1021/acsphotonics.7b01139.

[12] J. L. Montaño‐Priede , M. Zapata‐Herrera , R. Esteban , N. Zabala , and J. Aizpurua , “An Overview on Plasmon‐Enhanced Photoluminescence via Metallic Nanoantennas,” Nanophotonics 13, no. 26 (2024): 4771–4794, 10.1515/nanoph-2024-0463. PMC11614590 39640204

[13] G. Baffou , Thermoplasmonics: Heating Metal Nanoparticles Using Light (Cambridge University Press, 2017).

[14] M. Kim , J.‐H. Lee , and J.‐M. Nam , “Plasmonic Photothermal Nanoparticles for Biomedical Applications,” Advanced Science 6, no. 17 (2019): 1900471, 10.1002/advs.201900471. PMC6724476 31508273

[15] G. Baffou , F. Cichos , and R. Quidant , “Applications and Challenges of Thermoplasmonics,” Nature Materials 19, no. 9 (2020): 946–958, 10.1038/s41563-020-0740-6. 32807918

[16] B. Yang , C. Li , Z. Wang , and Q. Dai , “Thermoplasmonics in Solar Energy Conversion: Materials, Nanostructured Designs, and Applications,” Advanced Materials 34, no. 26 (2022): 2107351, 10.1002/adma.202107351. 35271744

[17] R. Verma , G. Sharma , and V. Polshettiwar , “The Paradox of Thermal vs. Non‐Thermal Effects in Plasmonic Photocatalysis,” Nature Communications 15, no. 1 (2024): 7974, 10.1038/s41467-024-51916-3. PMC11393361 39266509

[18] A. O. Govorov and H. H. Richardson , “Generating Heat With Metal Nanoparticles,” Nano Today 2, no. 1 (2007): 30–38, 10.1016/s1748-0132(07)70017-8.

[19] G. Baffou , R. Quidant , and F. J. García de Abajo , “Nanoscale Control of Optical Heating in Complex Plasmonic Systems,” ACS Nano 4, no. 2 (2010): 709–716, 10.1021/nn901144d. 20055439

[20] G. Baffou and R. Quidant , “Thermo‐Plasmonics: Using Metallic Nanostructures as Nano‐Sources of Heat,” Laser & Photonics Reviews 7, no. 2 (2013): 171–187, 10.1002/lpor.201200003.

[21] J. B. Herzog , M. W. Knight , and D. Natelson , “Thermoplasmonics: Quantifying Plasmonic Heating in Single Nanowires,” Nano Letters 14, no. 2 (2014): 499–503, 10.1021/nl403510u. 24382140

[22] A. P. Bell , J. A. Fairfield , E. K. McCarthy , et al., “Quantitative Study of the Photothermal Properties of Metallic Nanowire Networks,” ACS Nano 9, no. 5 (2015): 5551–5558, 10.1021/acsnano.5b01673. 25938797

[23] Q. Jiang , B. Rogez , J.‐B. Claude , G. Baffou , and J. Wenger , “Temperature Measurement in Plasmonic Nanoapertures Used for Optical Trapping,” ACS Photonics 6, no. 7 (2019): 1763–1773, 10.1021/acsphotonics.9b00519.

[24] S. Adhikari , P. Spaeth , A. Kar , M. D. Baaske , S. Khatua , and M. Orrit , “Photothermal Microscopy: Imaging the Optical Absorption of Single Nanoparticles and Single Molecules,” ACS Nano 14, no. 12 (2020): 16414–16445, 10.1021/acsnano.0c07638. PMC7760091 33216527

[25] S. Fahad , S. Li , Y. Zhai , C. Zhao , Z. Pikramenou , and M. Wang , “Luminescence‐Based Infrared Thermal Sensors: Comprehensive Insights,” Small 20, no. 3 (2024): 2304237, 10.1002/smll.202304237. 37679096

[26] X. Yu , S. Fan , B. Zhu , S. I. El‐Hout , J. Zhang , and C. Chen , “Recent Progress on Photothermal Nanomaterials: Design, Mechanism, and Applications,” Green Energy & Environment 10, no. 7 (2025): 1377–1436, 10.1016/j.gee.2024.09.002.

[27] Z. Chen , M. Chen , H. Yan , P. Zhou , and X. Chen , “Enhanced Solar Thermal Conversion Performance of Plasmonic Gold Dimer Nanofluids,” Applied Thermal Engineering 178 (2020): 115561, 10.1016/j.applthermaleng.2020.115561.

[28] L. Zundel , K. Malone , L. Cerdán , R. Martínez‐Herrero , and A. Manjavacas , “Lattice Resonances for Thermoplasmonics,” ACS Photonics 10, no. 1 (2023): 274–282, 10.1021/acsphotonics.2c01610. PMC10288824 37363634

[29] L. Khosravi Khorashad , L. V. Besteiro , Z. Wang , J. Valentine , and A. O. Govorov , “Localization of Excess Temperature Using Plasmonic Hot Spots in Metal Nanostructures: Combining Nano‐Optical Antennas With the Fano Effect,” Journal of Physical Chemistry C 120, no. 24 (2016): 13215–13226, 10.1021/acs.jpcc.6b03644.

[30] K. Li , M. I. Stockman , and D. J. Bergman , “Self‐Similar Chain of Metal Nanospheres as an Efficient Nanolens,” Physical Review Letters 91, no. 22 (2003): 227402, 10.1103/physrevlett.91.227402. 14683271

[31] J. A. Lloyd , S. H. Ng , A. C. Y. Liu , et al., “Plasmonic Nanolenses: Electrostatic Self‐Assembly of Hierarchical Nanoparticle Trimers and Their Response to Optical and Electron Beam Stimuli,” ACS Nano 11, no. 2 (2017): 1604–1612: pMID: 28165711, 10.1021/acsnano.6b07336. 28165711

[32] A. Sánchez‐Iglesias , N. Claes , D. M. Solís , et al., “Reversible Clustering of Gold Nanoparticles Under Confinement,” Angewandte Chemie International Edition 57, no. 12 (2018): 3183–3186, 10.1002/anie.201800736. PMC6468316 29417726

[33] K. L. Kelly , E. Coronado , L. L. Zhao , and G. C. Schatz , “The Optical Properties of Metal Nanoparticles: The Influence of Size, Shape, and Dielectric Environment,” Journal of Physical Chemistry B 107, no. 3 (2003): 668–677, 10.1021/jp026731y.

[34] K. A. Willets and R. P. Van Duyne , “Localized Surface Plasmon Resonance Spectroscopy and Sensing,” Annual Review of Physical Chemistry 58, no. 1 (2007): 267–297, 10.1146/annurev.physchem.58.032806.104607. 17067281

[35] L. Xin , M. Lu , S. Both , et al., “Watching a Single Fluorophore Molecule Walk Into a Plasmonic Hotspot,” ACS Photonics 6, no. 4 (2019): 985–993, 10.1021/acsphotonics.8b01737.

[36] A. Kuzyk , R. Jungmann , G. P. Acuna , and N. Liu , “DNA Origami Route for Nanophotonics,” ACS Photonics 5, no. 4 (2018): 1151–1163, 10.1021/acsphotonics.7b01580. PMC6156112 30271812

[37] J. L. Montaño‐Priede , A. Rao , A. Sánchez‐Iglesias , and M. Grzelczak , “Accelerated Design of Gold Nanoparticles With Enhanced Plasmonic Performance,” Science Advances 11, no. 33 (2025): eadx2299, 10.1126/sciadv.adx2299. PMC12356264 40815654

[38] G. Baffou , R. Quidant , and C. Girard , “Thermoplasmonics Modeling: A Green’s Function Approach,” Physical Review B: Condensed Matter 82, no. 16 (2010): 165424, 10.1103/physrevb.82.165424.

[39] U. Hohenester and A. Trügler , “MNPBEM—A Matlab Toolbox for the Simulation of Plasmonic Nanoparticles,” Computer Physics Communications 183, no. 2 (2012): 370–381, 10.1016/j.cpc.2011.09.009.

[40] P. B. Johnson and R. W. Christy , “Optical Constants of the Noble Metals,” Physical Review B: Condensed Matter 6, no. 12 (1972): 4370–4379, 10.1103/physrevb.6.4370.

[41] P. E. Stamatopoulou and C. Tserkezis , “Finite‐Size and Quantum Effects in Plasmonics: Manifestations and Theoretical Modelling [Invited],” Optical Materials Express 12, no. 5 (2022): 1869, 10.1364/ome.456407.

[42] W. Zhu , R. Esteban , A. G. Borisov , et al., “Quantum Mechanical Effects in Plasmonic Structures With Subnanometre Gaps,” Nature Communications 7, no. 1 (2016): 11495, 10.1038/ncomms11495. PMC4895716 27255556

[43] M. Urbieta , M. Barbry , Y. Zhang , et al., “Atomic‐Scale Lightning Rod Effect in Plasmonic Picocavities: A Classical View to a Quantum Effect,” ACS Nano 12, no. 1 (2018): 585–595, 10.1021/acsnano.7b07401. 29298379

[44] J. Cunha , T.‐L. Guo , G. Della Valle , A. N. Koya , R. Proietti Zaccaria , and A. Alabastri , “Controlling Light, Heat, and Vibrations in Plasmonics and Phononics,” Advanced Optical Materials 8, no. 24 (2020): 2001225, 10.1002/adom.202001225.

[45] G. Baffou and H. Rigneault , “Femtosecond‐Pulsed Optical Heating of Gold Nanoparticles,” Physical Review B: Condensed Matter 84, no. 3 (2011): 035415, 10.1103/physrevb.84.035415.

[46] D. A. G. Bruggeman , “Berechnung Verschiedener Physikalischer Konstanten Von Heterogenen Substanzen. I. Dielektrizitätskonstanten Und Leitfähigkeiten der Mischkörper Aus Isotropen Substanzen,” Annals of Physics 416, no. 7 (1935): 636–664, 10.1002/andp.19354160705.

[47] G. Wypych , Handbook of Fillers, G. Wypych , ed. 4th ed. (ChemTec Publishing, 2016), 13–266: ISBN 978‐1‐895198‐91‐1.

