# Supplementary material for: Controlling Nanoscale Heat With Optically Coupled Plasmonic Systems
Source: Nanophotonics. 2026 Jun 13;15(12):e70167. doi: 10.1002/nap2.70167 (PMC13642632; doi:10.1002/nap2.70167)
Supplement: Supplementary file 1 [file NAP2-15-e70167-s001.pdf]

Controlling Nanoscale Heat with Optically Coupled Plasmonic Systems

Supporting Information

José Luis Montaño-Priede[*,†] and Marek Grzelczak[*,†,‡]

†*Centro de Física de Materiales (CFM-MPC), CSIC-UPV/EHU, Paseo de Manuel Lardizabal 5, 20018 Donostia - San Sebastián, Spain*

‡*Donostia International Physics Center (DIPC), Paseo de Manuel Lardizabal 4, 20018 Donostia - San Sebastián, Spain*

E-mail: joseluis.montano@ehu.eus; marek.g@csic.es

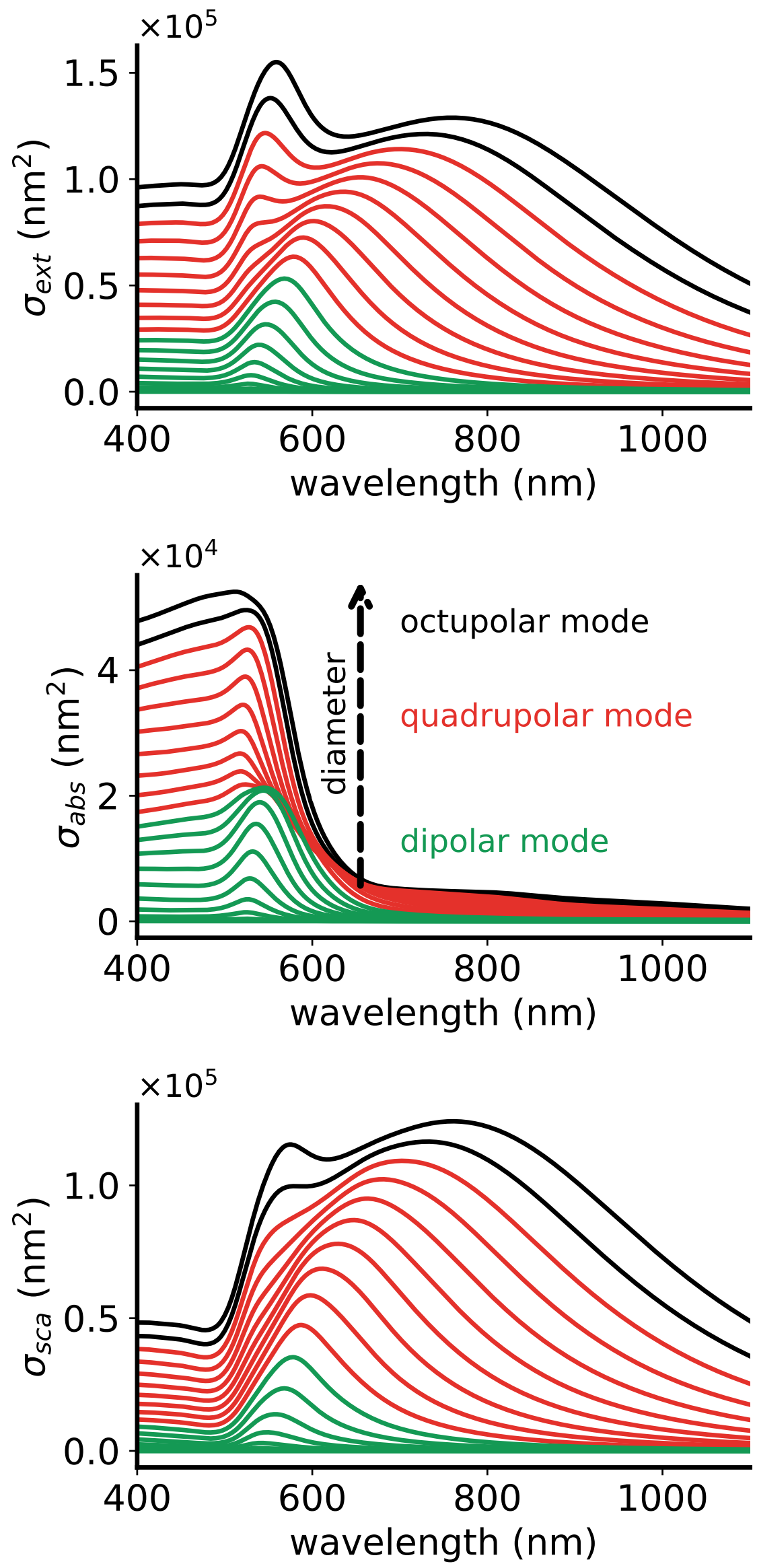

Figure S1: Extinction (σ_{ext}), absorption (σ_{abs}), and scattering (σ_{sca}) cross-section spectra of a single gold nanoparticle with diameter varied from 10 to 200 nm. The color of the spectra (green, red, black) denotes the dominant contributing plasmon resonance: dipole, quadrupole, and octupole, respectively.

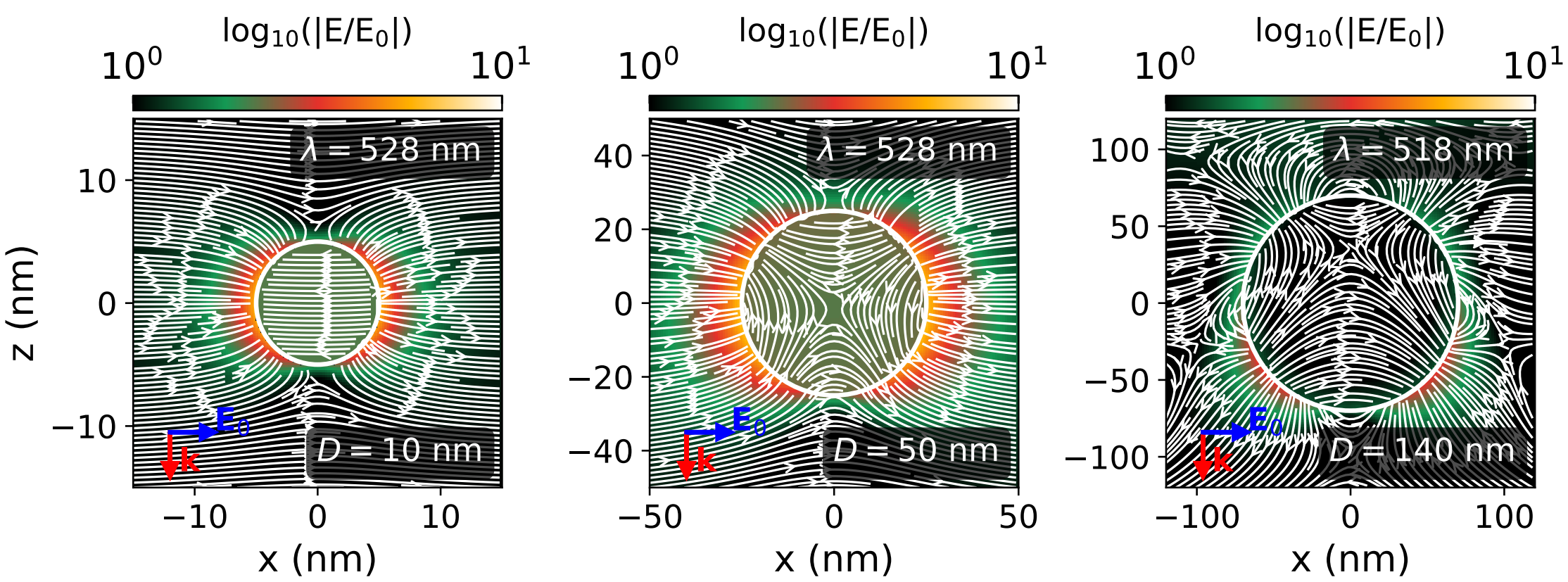

Figure S2: Electric field enhancement ($\log|\mathbf{E}/\mathbf{E}_0|^2$) and electric field lines in the xz-plane for single gold nanoparticles with diameters of 10 nm, 50 nm, and 140 nm. The white circle represents the nanoparticle boundary. The incident wave vector ($\mathbf{k}$) and incident electric field ($\mathbf{E}_0$) directions are indicated.

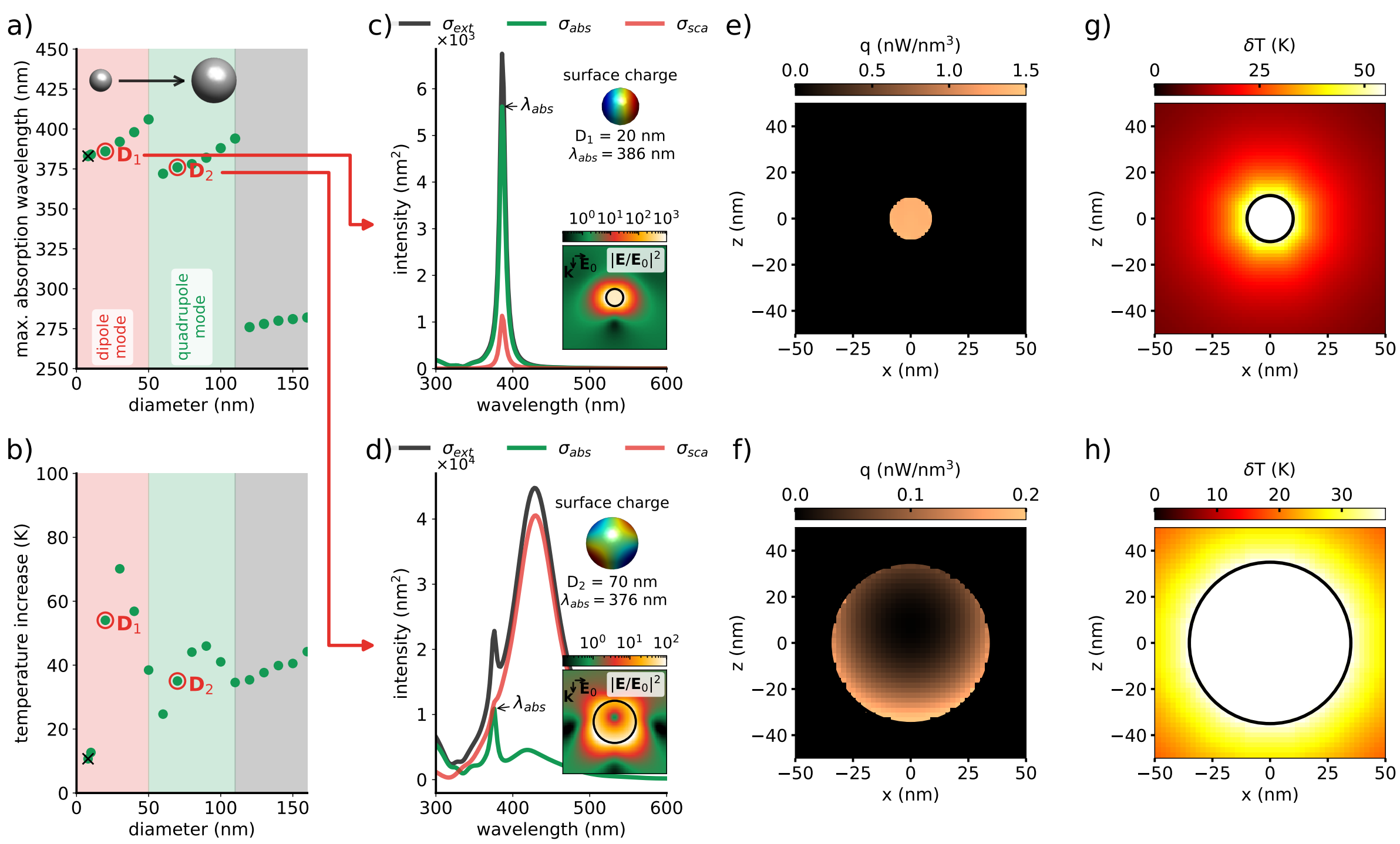

Figure S3: Thermoplasmonic response of a single Ag nanoparticle: diameter dependence. (a) Wavelength of the maximum absorption cross-section peak (λ_{abs}^{max}) as a function of nanoparticle diameter. (b) Temperature increase (δT) at λ_{abs}^{max} as a function of nanoparticle diameter, excitation irradiance was 10^9 W/m^2. (c, d) Spectra of the extinction, absorption, and scattering cross-sections for Ag nanoparticles with 20 nm (c) and 70 nm (d) diameters. Insets show the corresponding surface charge distribution and the electric field map at λ_{abs}^{max}. (e, f) Heat power density (q) maps for the AgNPs with 20 nm (e) and 70 nm (f) diameters. (g, h) Temperature increase for the Ag nanoparticles with 20 nm (g) and 70 nm (h) diameters. The results for an 8-nm NP (marked with a black cross in (a) and (b)) yield δT = 10.7 K, which is used as the reference to calculate the Photothermal Focusing Factor for the nanolens (see Table S1).

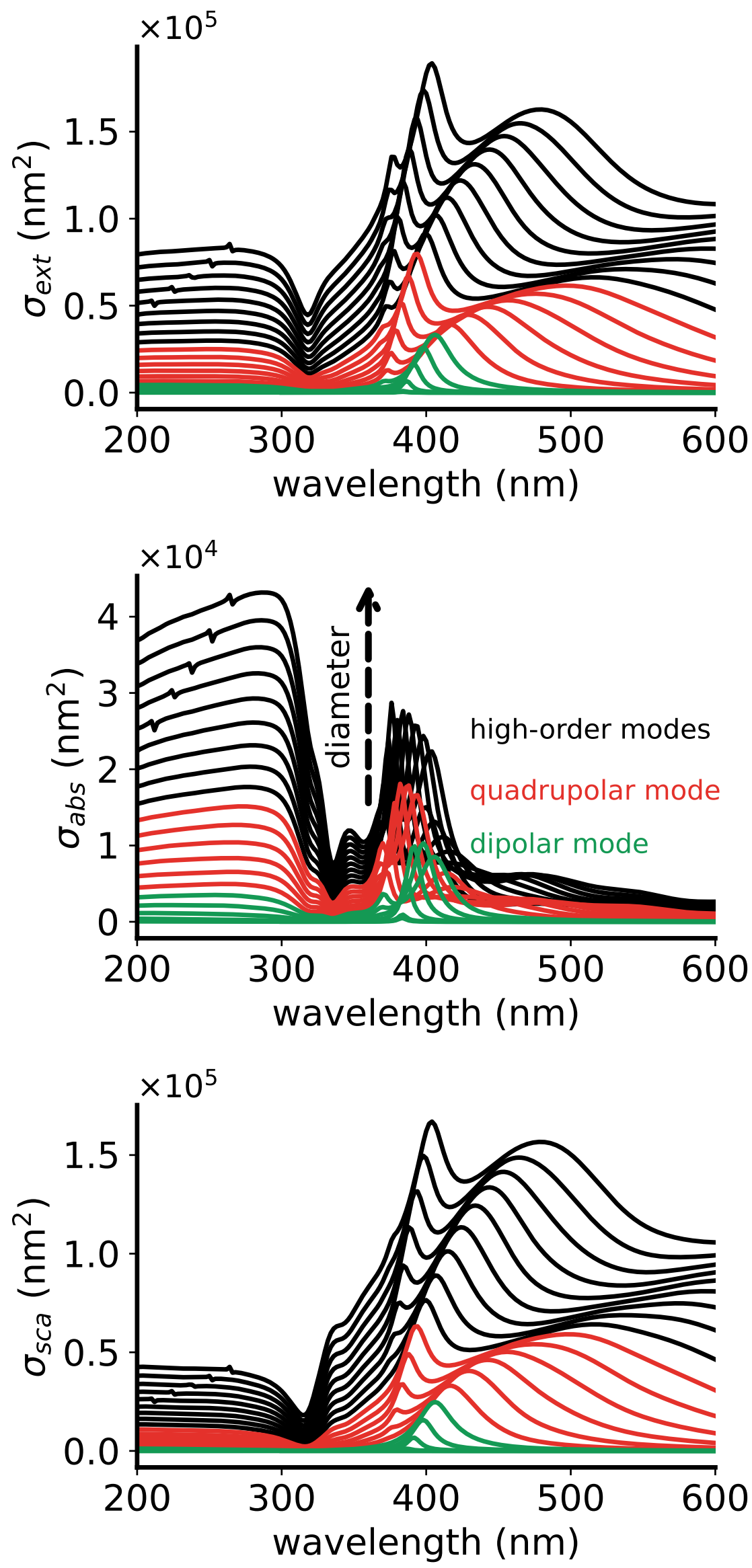

Figure S4: Extinction, absorption, and scattering cross-section spectra of a single silver nanoparticle with diameter varied from 10 to 200 nm. The color of the spectra (green, red, black) denotes the dominant contributing plasmon resonance: dipole, quadrupole, and higher-order modes, respectively.

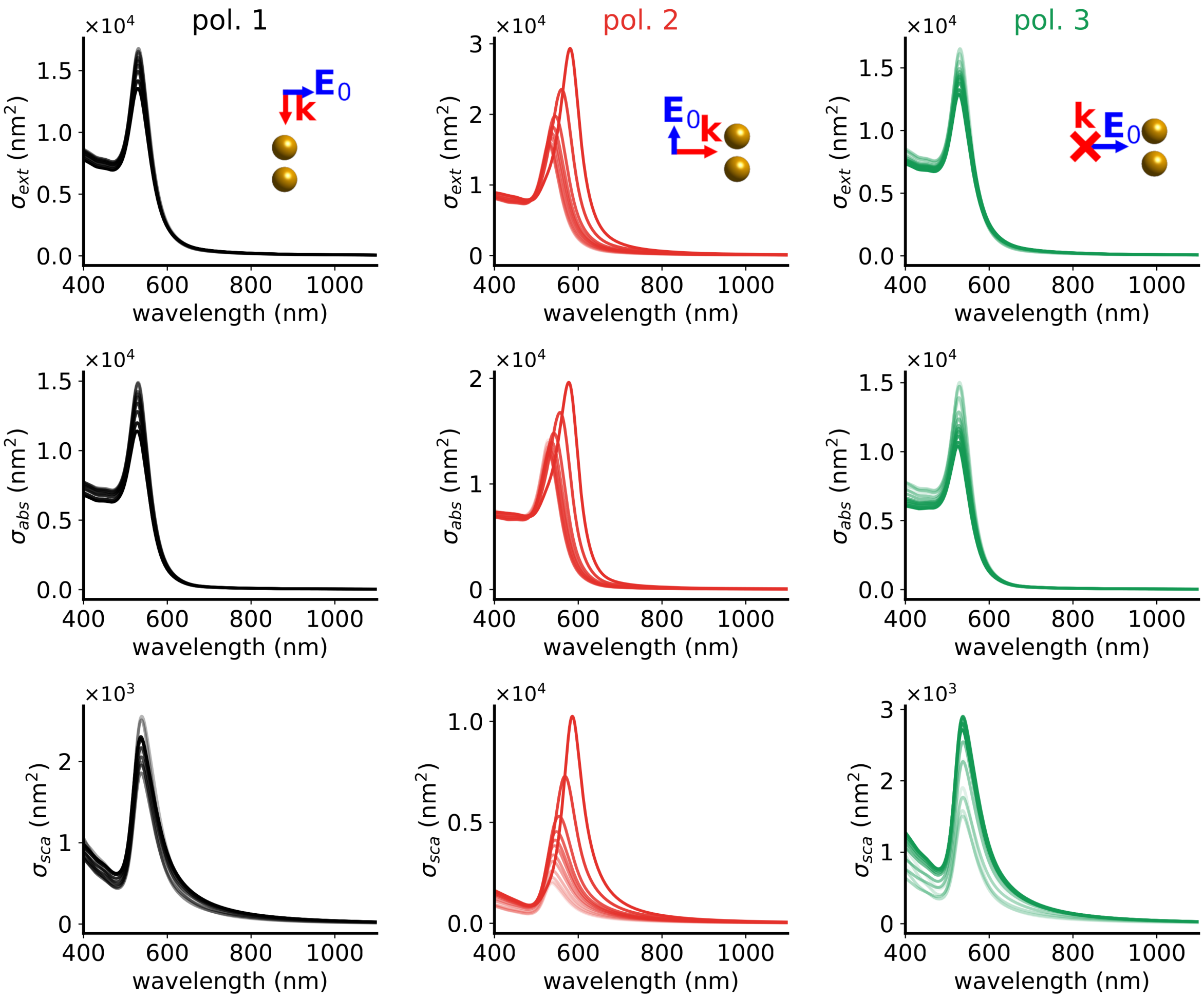

Figure S5: Extinction, absorption, and scattering cross-section spectra of a gold nanoparticle dimer under three orthogonal incident polarizations: transverse to the dimer axis (pol. 1, black), longitudinal along the dimer axis (pol. 2, red), and at 90^0 orientation to the axis (pol. 3, green). Each nanoparticle has a diameter of 50 nm, with an interparticle gap varied from 1 to 500 nm (spectrum color saturation from darker to lighter).

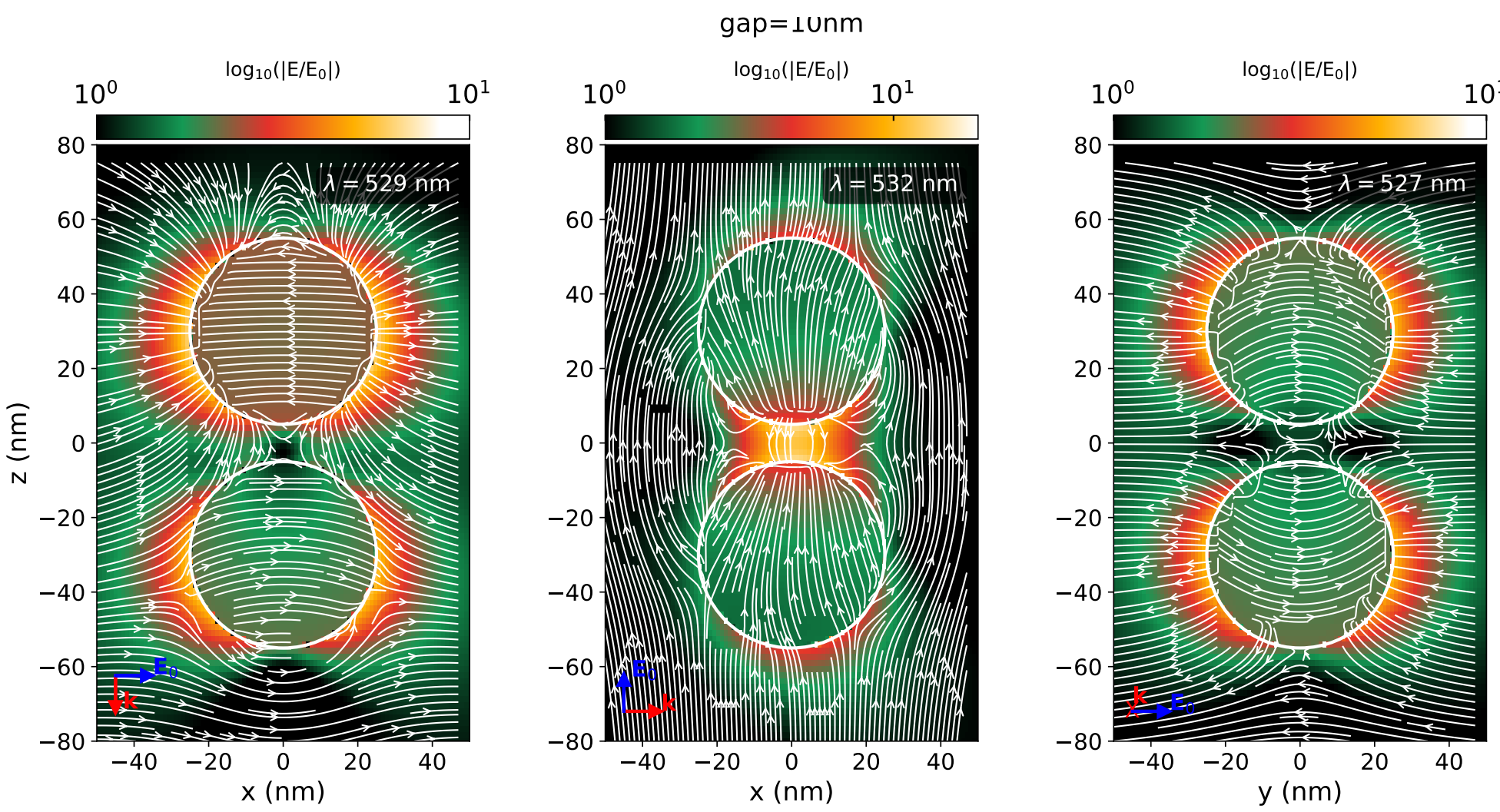

Figure S6: Electric field enhancement ($\log|\mathbf{E}/\mathbf{E}_0|^2$) and electric field lines in the plane of excitation for a dimer with a 10-nm gap under three incident polarizations. Each nanoparticle has a diameter of 50 nm. White circles in indicate nanoparticle boundaries, while red and blue arrows show the incident wave vector ($\mathbf{k}$) and incident electric field ($\mathbf{E}_0$) directions, respectively.

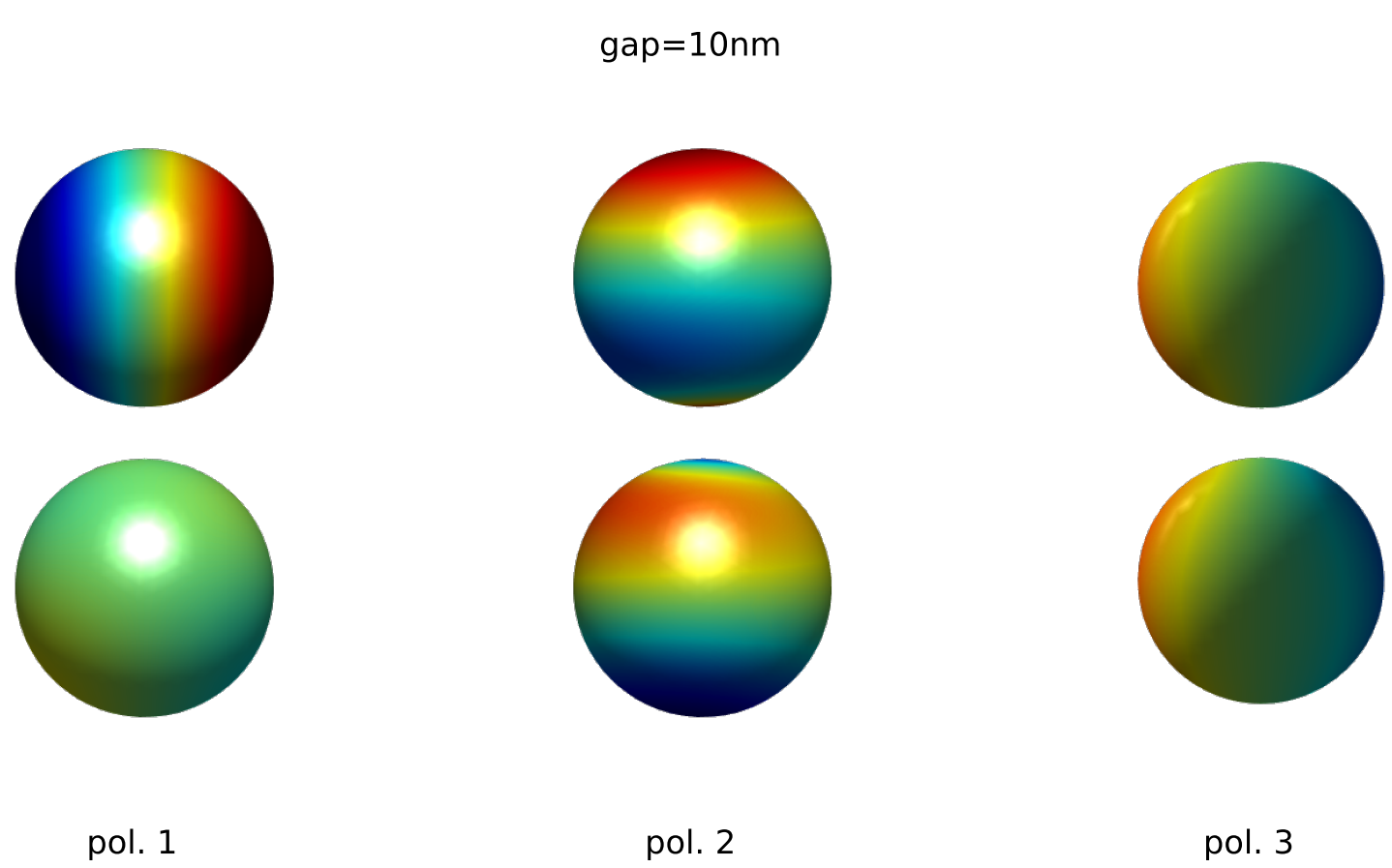

Figure S7: Surface charge density distribution for a dimer with a 10-nm gap under three incident polarizations. Each nanoparticle has a diameter of 50 nm. For polarization 3, the dimer is rotated 90^0 around the z-axis to visualize the charge distribution.

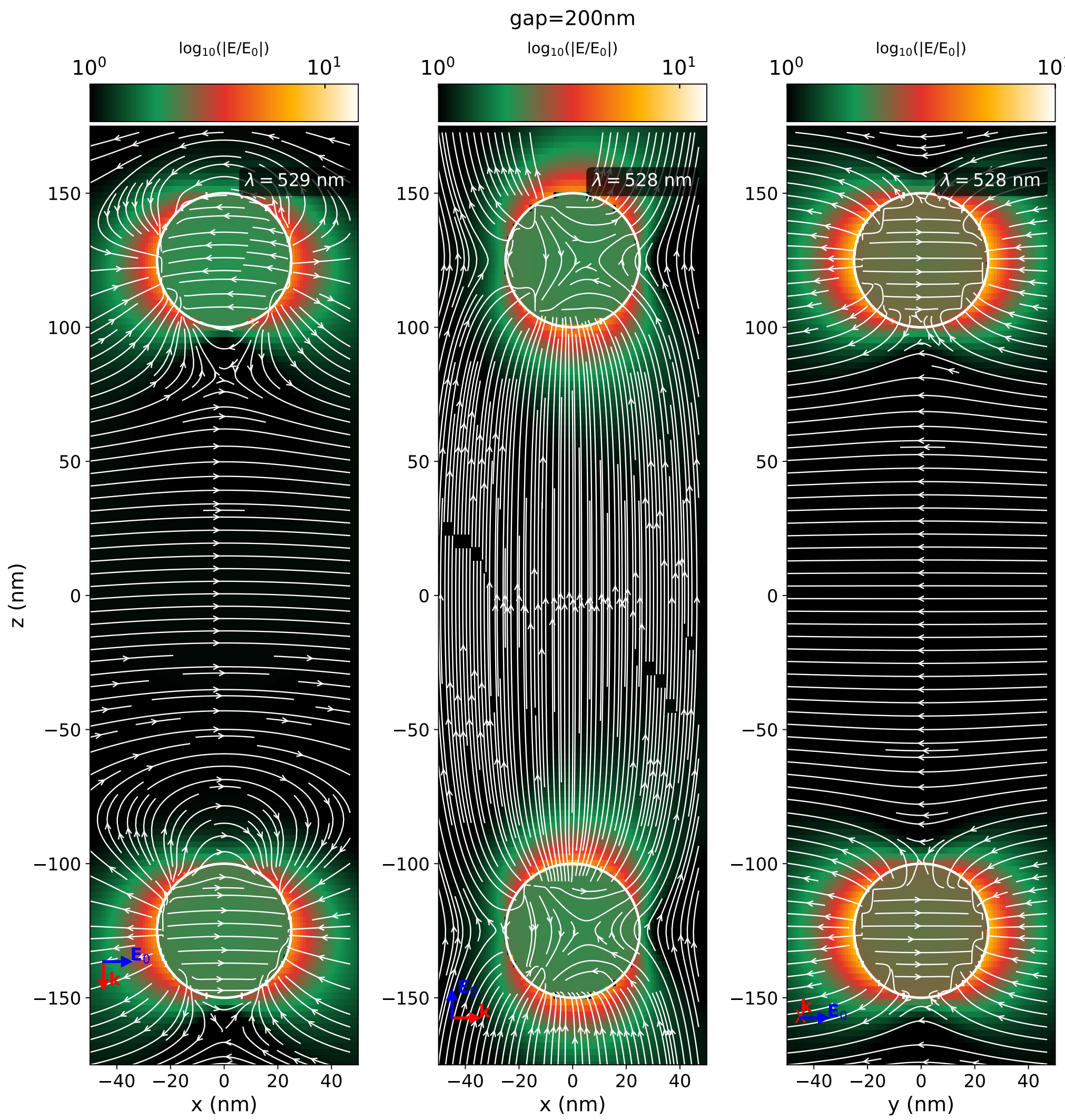

Figure S8: Electric field enhancement ($\log|\mathbf{E}/\mathbf{E}_0|^2$) and electric field lines in the plane of excitation for a dimer with a 200-nm gap under three incident polarizations. Each nanoparticle has a diameter of 50 nm. White circles indicate nanoparticle boundaries, while red and blue arrows show the incident wave vector ($\mathbf{k}$) and incident electric field ($\mathbf{E}_0$) directions, respectively.

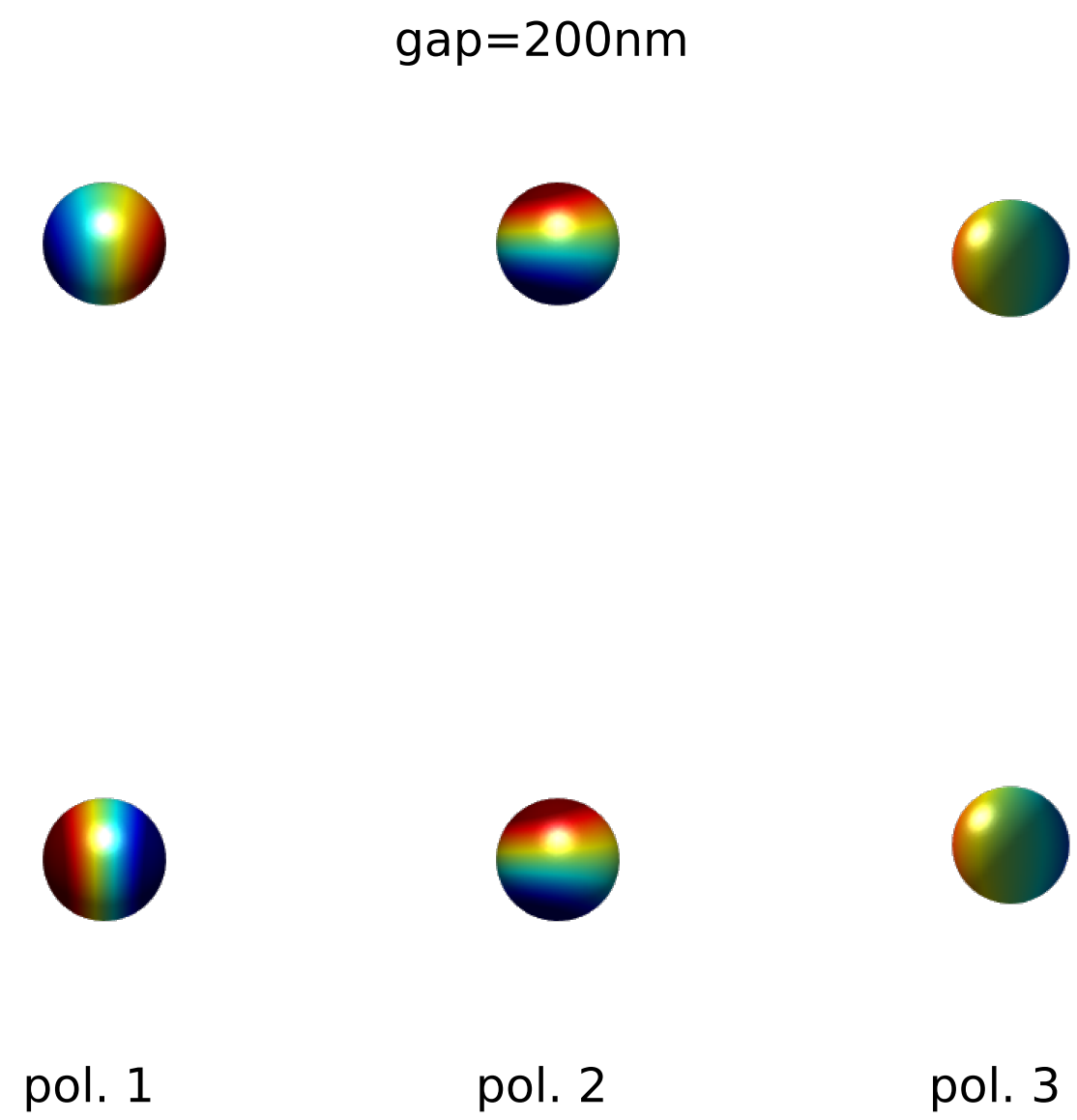

Figure S9: Surface charge density distribution for a dimer with a 200-nm gap under three incident polarizations. Each nanoparticle has a diameter of 50 nm. For polarization 3, the dimer is rotated 90^0 around the z-axis to visualize the charge distribution.

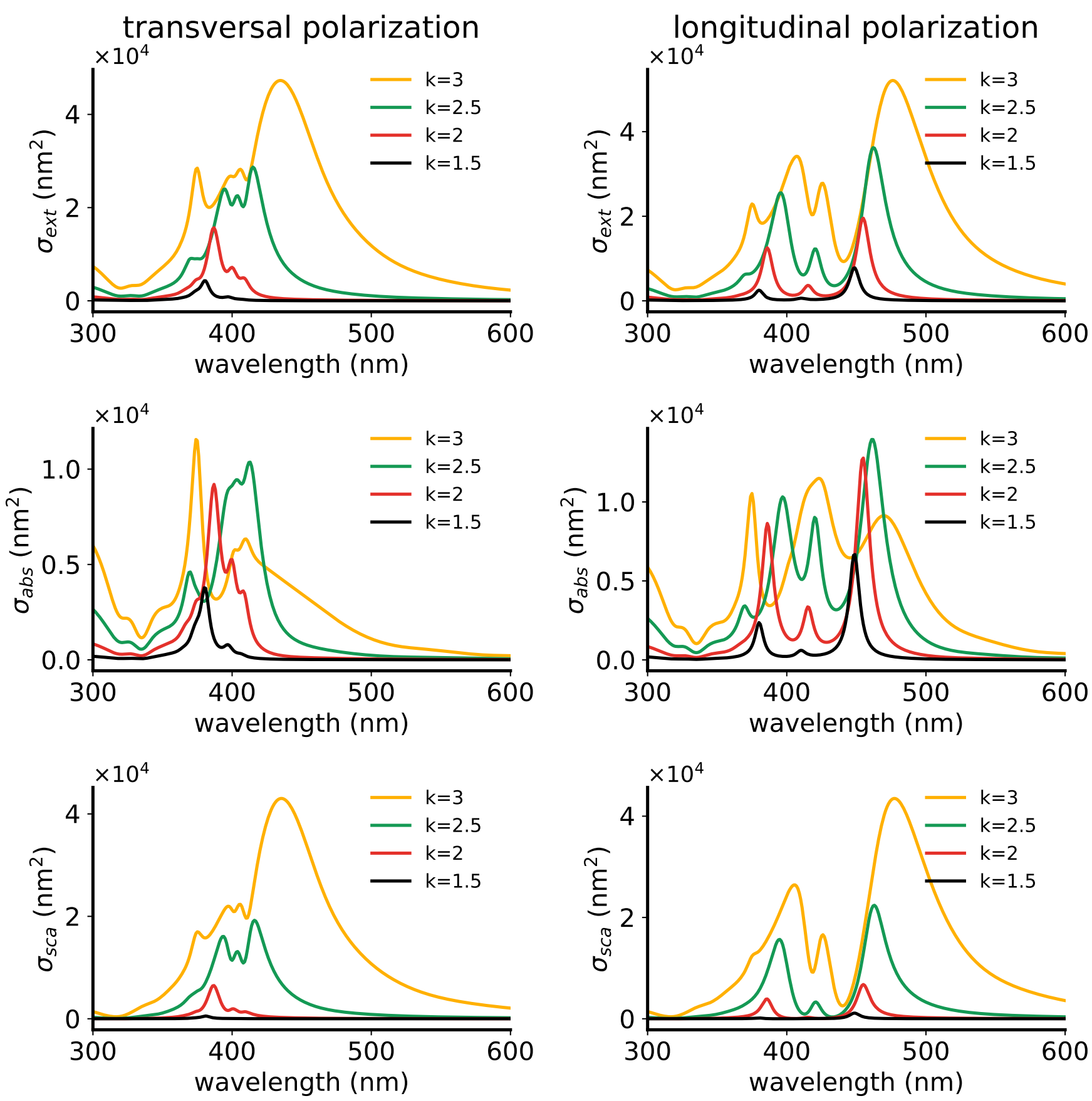

Figure S10: Optical cross-section spectra of a silver nanolens trimer. The structure consists of three nanoparticles with diameters and gaps scaled by a geometrical self-factor κ (1.5, 2, 2.5, 3), see scheme in Figure 3b in the main text. Spectra are shown for two incident polarizations: transverse (solid lines) and longitudinal (dashed lines) to the trimer axis.

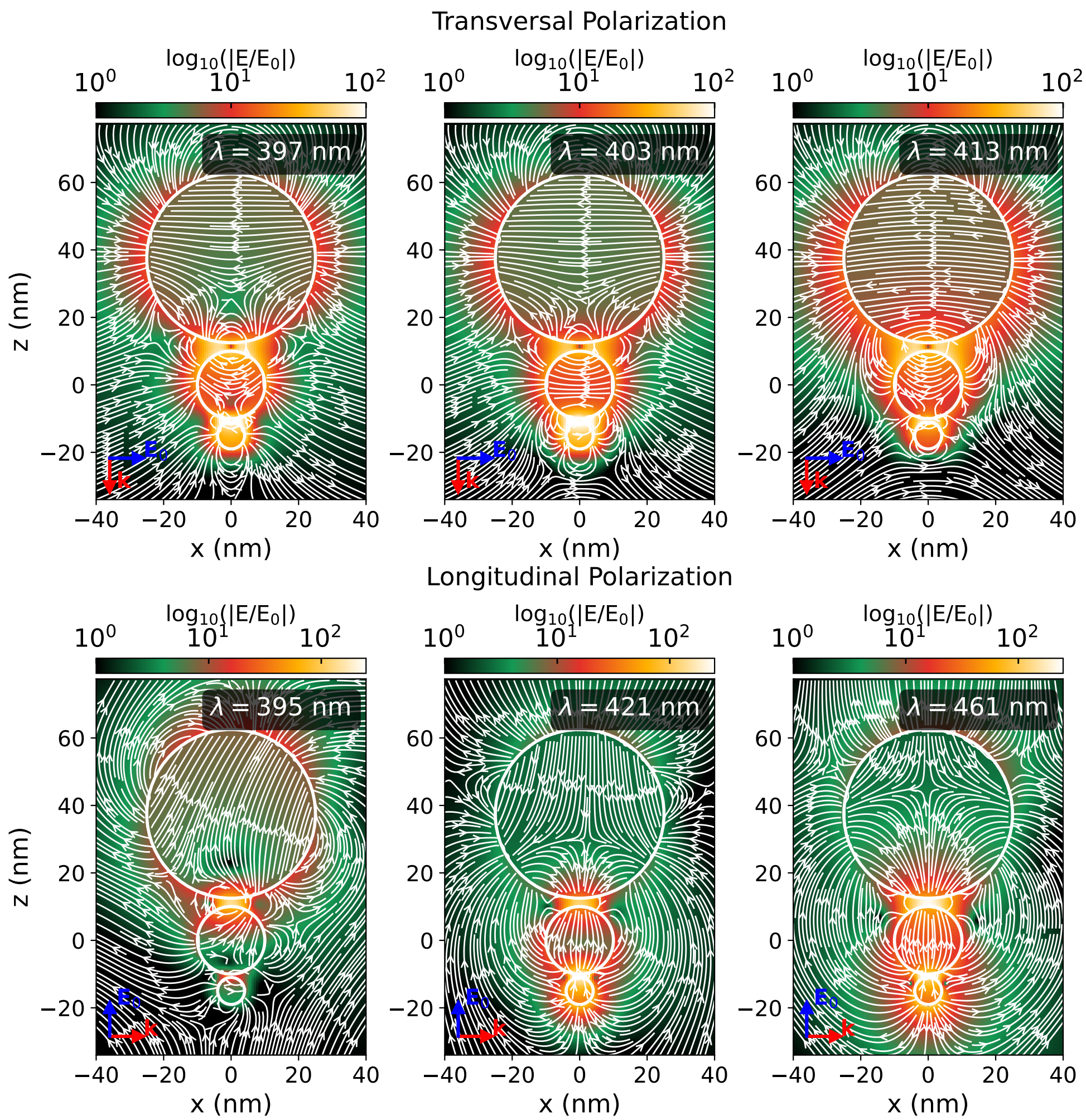

Figure S11: Electric field enhancement and electric field lines for a silver nanolens trimer with $\kappa = 2.5$. The map shows $\log|\mathbf{E}/\mathbf{E}_0|^2$ in the xz-plane at the dominant absorption peaks for transverse and longitudinal incident polarizations. White circles indicate nanoparticle boundaries, while arrows show the incident wave vector ($\mathbf{k}$) and incident electric field ($\mathbf{E}_0$) directions.

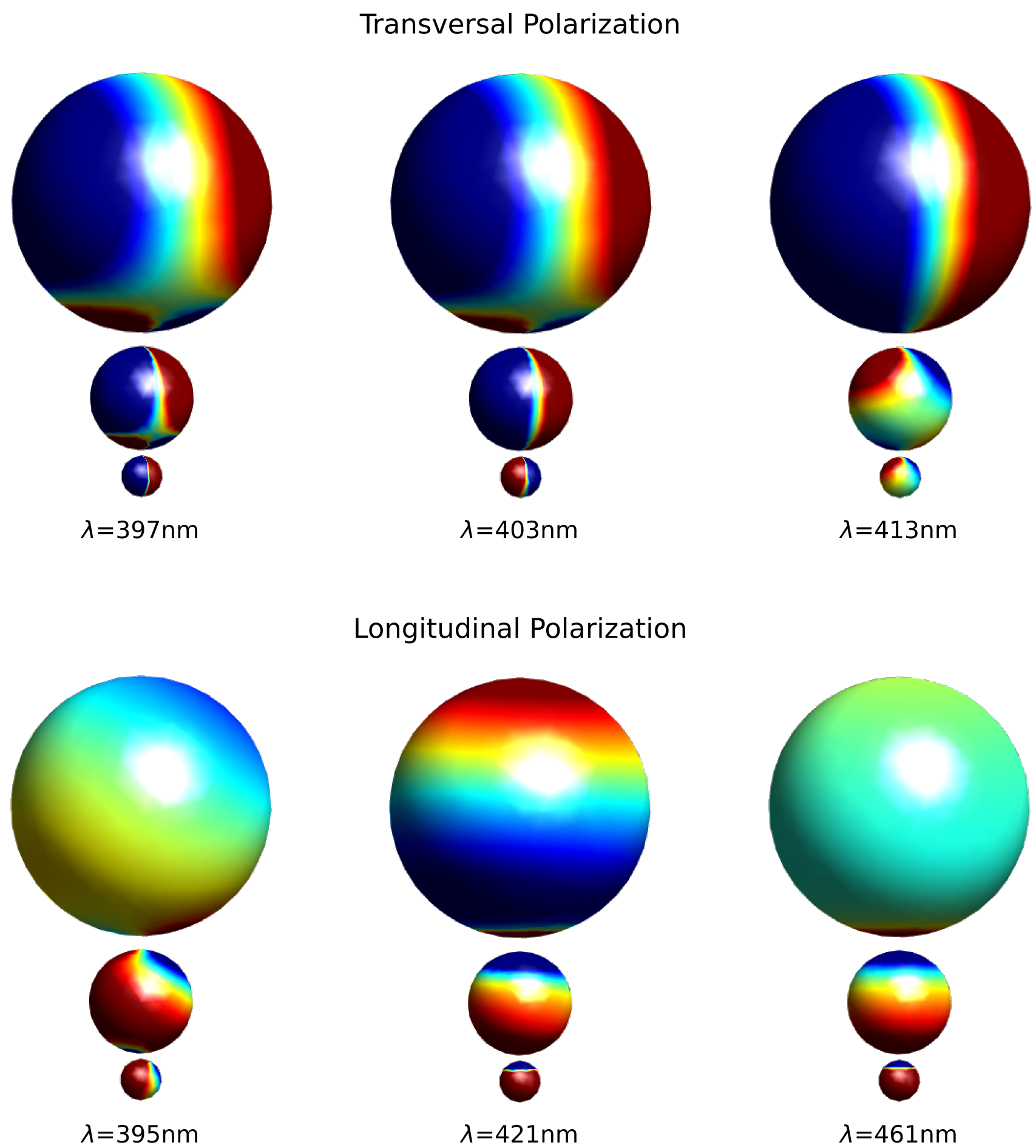

Figure S12: Surface charge distribution of the silver nanolens trimer with $\kappa = 2.5$ at the dominant absorption peaks. The top and bottom rows correspond to transverse and longitudinal incident polarizations, respectively. Each column shows the charge distribution for a specific resonant mode, ordered by increasing wavelength. Red and blue colors indicate positive and negative induced charge densities.

Table S1: Photothermal Focusing Factor (PFF) for the silver nanolens trimer. For each self-similar factor κ, polarization, and absorption peak, the table provides the temperature increase of the smallest (8 nm) nanoparticle δT(D3) and the PFF, defined as the ratio of δT(D3) to that of a single 8 nm Ag sphere ($\delta T = 10.7$ K), see Eq. 16 in the main document.

κ	Polarization	Absorption peak	δT(D3) [K]	PFF
1.5	Transverse	$\lambda_{abs,1}$	3.0	0.3
		$\lambda_{abs,2}$	8.4	0.8
		$\lambda_{abs,3}$	28.2	2.6
	Longitudinal	$\lambda_{abs,1}$	89.2	8.3
		$\lambda_{abs,2}$	7.5	0.7
		$\lambda_{abs,3}$	16.2	1.5
2.0	Transverse	$\lambda_{abs,1}$	23.6	2.2
		$\lambda_{abs,2}$	51.2	4.8
		$\lambda_{abs,3}$	50.7	4.7
	Longitudinal	$\lambda_{abs,1}$	140.2	13.1
		$\lambda_{abs,2}$	44.5	4.2
		$\lambda_{abs,3}$	41.2	3.9
2.5	Transverse	$\lambda_{abs,1}$	50.3	4.7
		$\lambda_{abs,2}$	86.0	8.0
		$\lambda_{abs,3}$	62.9	5.9
	Longitudinal	$\lambda_{abs,1}$	126.3	11.8
		$\lambda_{abs,2}$	122.6	11.5
		$\lambda_{abs,3}$	35.9	3.4
3.0	Transverse	$\lambda_{abs,1}$	9.2	0.9
		$\lambda_{abs,2}$	34.3	3.2
		$\lambda_{abs,3}$	40.3	3.8
	Longitudinal	$\lambda_{abs,1}$	55.0	5.1
		$\lambda_{abs,2}$	110.7	10.3
		$\lambda_{abs,3}$	25.5	2.4

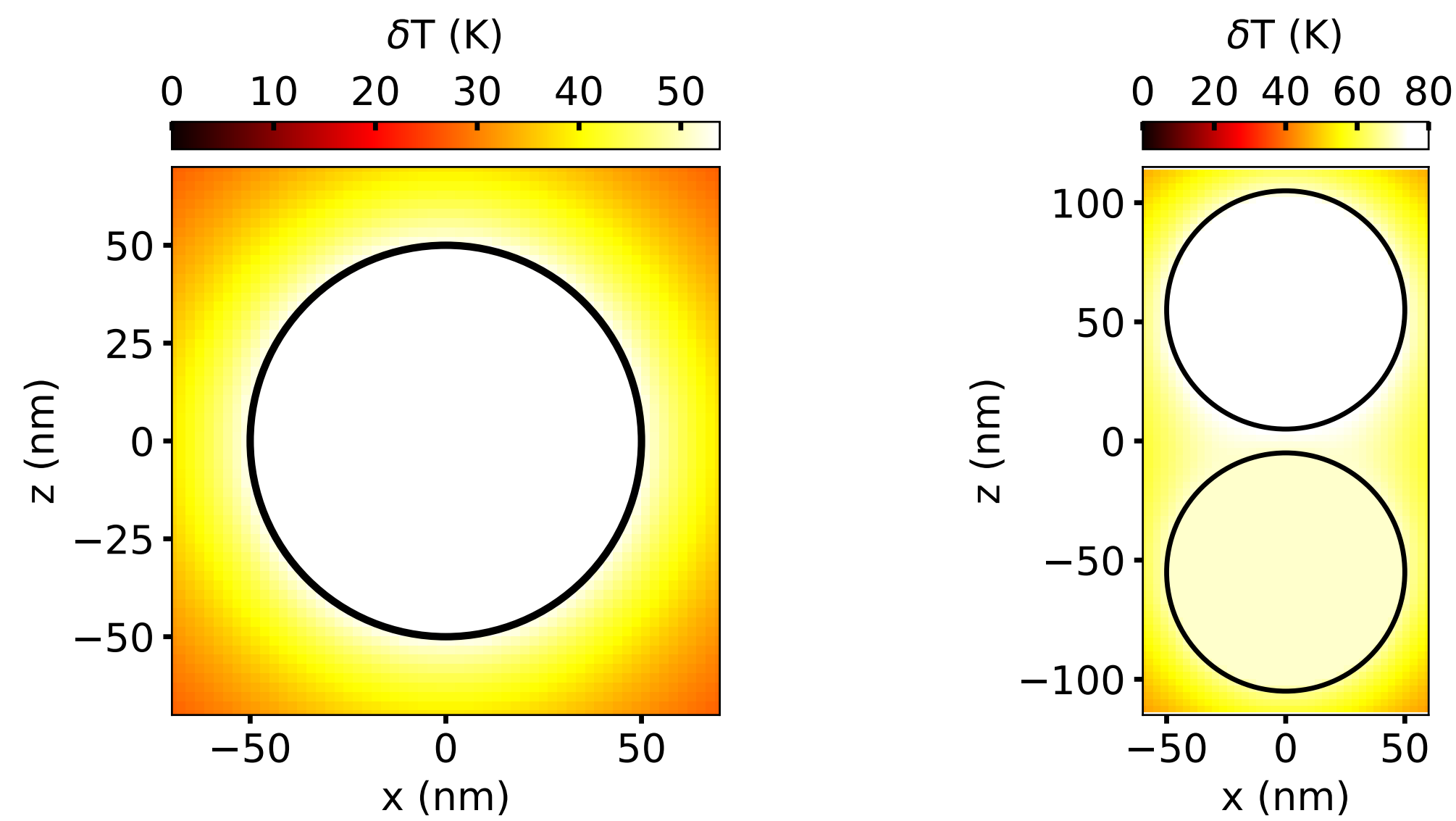

(a) (b)

Figure S13: Temperature increase of (a) single 100-nm AuNP and (b) a 100-nm-AuNP dimer with a 5-nm gap under 45° incident excitation angle, both in water. The results obtained hear show excellent agreement with those reported in Ref. 1 (their Fig. 1 and Fig. 4, respectively).

References

(1) Baffou, G.; Quidant, R.; García de Abajo, F. J. Nanoscale Control of Optical Heating in Complex Plasmonic Systems. *ACS Nano* **2010**, *4*, 709–716.
